# Estimating genetics of body dimensions and activity levels in pigs using automated pose estimation

**DOI:** 10.1038/s41598-022-19721-4

**Published:** 2022-09-13

**Authors:** Wim Gorssen, Carmen Winters, Roel Meyermans, Rudi D’Hooge, Steven Janssens, Nadine Buys

**Affiliations:** 1 Center for Animal Breeding and Genetics, Department of Biosystems, KU Leuven, Kasteelpark Arenberg 30, Box 2472, 3001 Leuven, Belgium; 2 Laboratory for Biological Psychology, KU Leuven, Tiensestraat 102, Box 3714, 3000 Leuven, Belgium

**Keywords:** Animal breeding, Behavioural genetics, Genetics, Machine learning

## Abstract

Pig breeding is changing rapidly due to technological progress and socio-ecological factors. New precision livestock farming technologies such as computer vision systems are crucial for automated phenotyping on a large scale for novel traits, as pigs’ robustness and behavior are gaining importance in breeding goals. However, individual identification, data processing and the availability of adequate (open source) software currently pose the main hurdles. The overall goal of this study was to expand pig weighing with automated measurements of body dimensions and activity levels using an automated video-analytic system: DeepLabCut. Furthermore, these data were coupled with pedigree information to estimate genetic parameters for breeding programs. We analyzed 7428 recordings over the fattening period of 1556 finishing pigs (Piétrain sire x crossbred dam) with two-week intervals between recordings on the same pig. We were able to accurately estimate relevant body parts with an average tracking error of 3.3 cm. Body metrics extracted from video images were highly heritable (61–74%) and significantly genetically correlated with average daily gain (r_g_ = 0.81–0.92). Activity traits were low to moderately heritable (22–35%) and showed low genetic correlations with production traits and physical abnormalities. We demonstrated a simple and cost-efficient method to extract body dimension parameters and activity traits. These traits were estimated to be heritable, and hence, can be selected on. These findings are valuable for (pig) breeding organizations, as they offer a method to automatically phenotype new production and behavioral traits on an individual level.

## Introduction

Pork already accounts for almost 40% of meat production worldwide, but its global demand is expected to grow even further^1^. Increasingly, pig farming is facing challenges such as environmental sustainability, animal welfare, farming efficiency and global competition. Innovations in genetics are considered crucial to solve these issues^2,3^. Traits related to robustness and resilience are now as relevant to animal breeding programs as those related to (re)production^2^. There is obviously a need for precision livestock farming technologies to optimize high-throughput pig phenotyping, which to this day remains difficult, labor intensive and costly^4^.

Computer vision systems (CVS) could enable non-invasive phenotyping of body composition, behavior and physical abnormalities in pigs, but there have been few practical applications of automated scoring of morphological and/or behavioral traits. Fernandes et al.^5^ used top-view 3D-cameras to predict body weight, muscle depth and back fat in finishing pigs. Other studies used CVS to assess body positions and posture^6^, aggression^7^, sow behavior^8^ and feeding^9,10^. Only a few studies analyzed the genetics of these phenotypes^4^. In beef cattle, heritability and genetic correlations were estimated using image analysis for carcass traits^11^. Also, genetic parameters for coat color and conformational traits have been estimated in dairy cattle using images from web catalogs^12^.

Morphological traits of pigs such as body length, width and height are reported to be moderately to highly heritable, with heritability (h^2^) estimates ranging from 20 to 60%^13–15^. Behavioral traits have been shown to be heritable as well, but h^2^ estimates differ considerably^3^. These estimates were most often based on manual scoring of behavioral traits that are susceptible to observer bias^16^. For example, activity scores during weighing were estimated to be low to moderately heritable (h^2^ = 10–23%), although there are environmental confounds that influenced these estimates^3,17–19^. Notably, Ott et al.^16^ showed that CVS activity scores were highly correlated with human observations (r = 0.92). Other studies related changes in activity scores in the pen to tail biting, infections^16,20–22^, or residual feed intake^23^. Incidentally, the relationship between activity during weighing and pen aggression was low to moderate (r = 0.15–0.60)^3,24^.

Automatic identification of animals remains a main challenge for CVS applications in phenotype-genotype analysis^4,25,26^. Also, data processing and availability of adequate software are issues^2,4,25^. Recent advances in motion capture and deep-learning tools now allow researchers to extract detailed behavioral variables from recorded videos without specialized and excessive recording hardware or setups^27^. These tools reach human-like accuracy and even outperform manual scoring^28,29^.

The current study describes a method that uses open source DeepLabCut (DLC) software^27^, a Python-based^30^ Graphical User Interface for markerless pose estimation Convolutional Neural Network (CNN)-based technology. DLC enables to create reliable and transferable tracking models^29^ to quantify various (behavioral) traits with limited observer influence. We combine classical weighing of pigs with video analysis to estimate new phenotypes. We obtain measurements of body length, hip and shoulder width and surface area, as well as activity scores of pigs during weighing. Based on these body composition and activity traits, we estimated genetic parameters such as heritability and genetic correlations in a population of slaughter pigs with known pedigree. All annotated videos and tracking models are available on: 10.17605/OSF.IO/QKW5Y.

## Methods

### Ethics statement

All experimental procedures were approved by the Animal Ethics Committee of KU Leuven (P004/2020), in accordance with European Community Council Directive 86/609/EEC, the ARRIVE guidelines and the ILAR Guide to the Care and Use of Experimental Animals. Researchers obtained informed consent for publication from all identifiable persons to display and reuse videos.

### Animals and housing

The study was carried out on 794 female and 746 castrated male Piétrain x PIC Camborough pigs (Vlaamse Piétrain Fokkerij, Belgium; offspring from 73 different sires and 204 dams), which had a mean age of 83.4 (± 2.2) days and a mean weight of 30.6 (± 5.1) kg at the start of the experiment. Observations were made during the fattening period which could span up to 120 days and ended when pigs reached a body weight of approximately 115 kg. Per sire, a median of 26 crossbred piglets (full-sibs and half-sibs from the same Piétrain sire) were allocated in equal numbers to two identical pens in mixed-sex groups. The pig building (experimental farm, located in Belgium) consisted of seventeen identical compartments with eight semi-slatted pens (2.5 m × 4.0 m) per compartment and on average thirteen pigs per pen (0.77m^2^ per pig). Food and water were provided ad-libitum in each pen throughout, from one trough and one nipple drinker.

### Data collection

Pigs were weighed individually over their fattening period every two weeks from January to July 2021. Pen-by-pen, all individuals were driven to the stable’s central hallway, after which pigs were weighed sequentially. Weighing was carried out between 08:00 a.m. and 16:00 p.m. and was video-recorded. All piglets were weighed for the first at thirteen days after arrival at the fattening farm. For practical limitations, only one out of two pens per sire was hereafter selected for subsequent follow-up. All 1556 pigs were weighed up to eight times, resulting in a total of 7428 records.

Additionally, each pig was scored manually during weighing on the following physical abnormalities: ear swellings or hematomas (0 = none, 1 = one ear, 2 = both ears); the presence and size of umbilical hernia (0 = not present, 1 = present); ear biting wounds (0 = none, 1 = one ear, 2 = both ears) and tail biting wounds (0 = none, 1 = small scratches, 3 = bloody and/or infected tail; Additional File 1). All recordings were collected by the same trained professional. Lean meat percentage was recorded individually at the slaughterhouse of the Belgian Pork Group in Meer (Belgium) using AutoFom III™ (Frontmatec, Smoerum A/S, Denmark)^31^. Feed intake was measured at the pen level.

### Experimental setup and equipment

**Figure 1:**
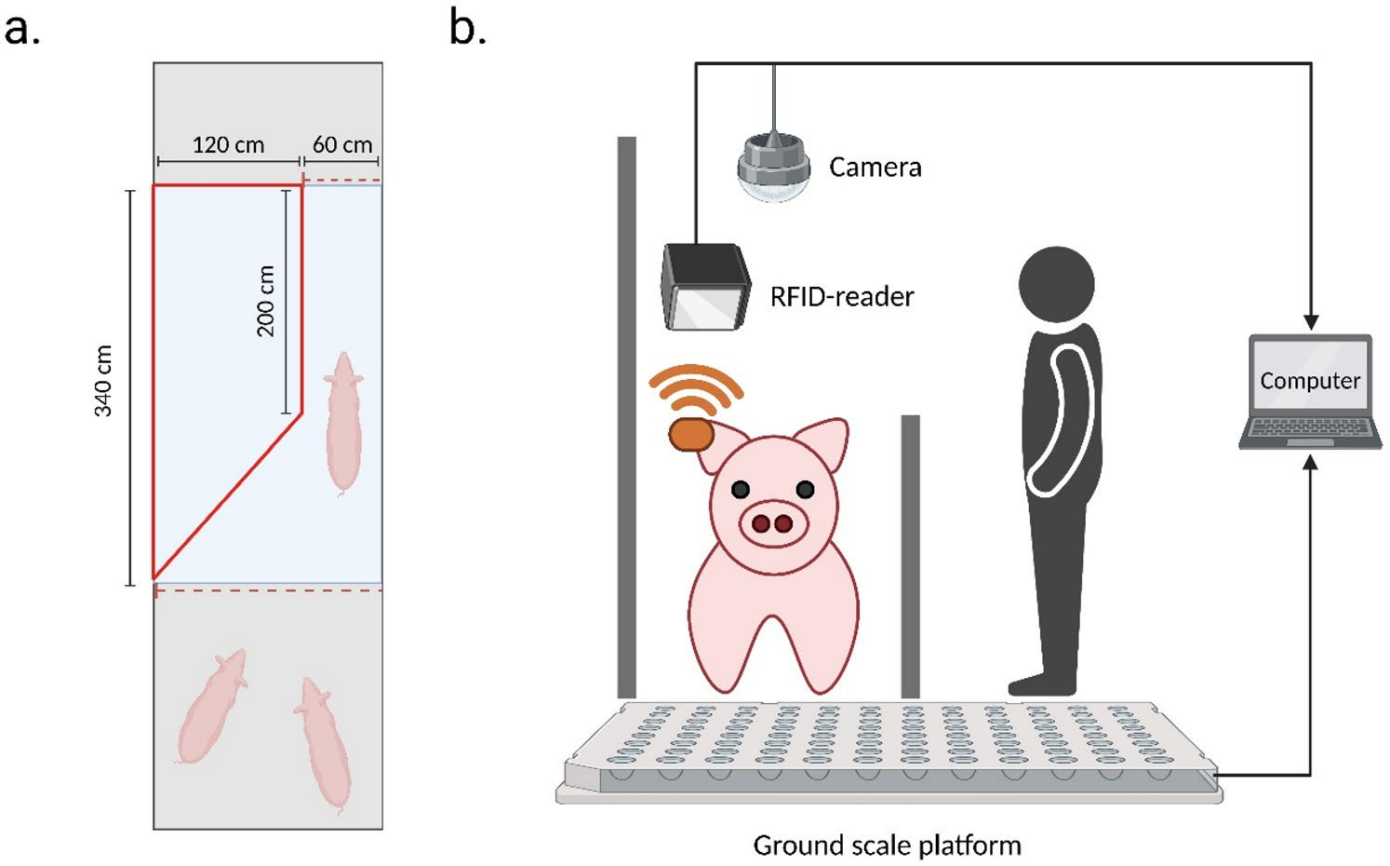
Experimental setup (created with BioRender.com). (**a**) Schematic top view diagram of the experimental setup used in this study in the center hallway of the pig building. The blue area indicates the ground scale platform with a wooden aisle (in red). The red dashed lines indicate gates to regulate individual pig passage. (**b**) Schematic side view diagram of the experimental setup.

The walk-through pig weighing setup consisted of a ground scale weighing platform, a radio frequency identification (RFID) reader, a video camera and a computer (Fig. 1). The ground scale platform (3.4 m × 1.8 m) had an accuracy of ± 0.5 kg (T.E.L.L. EAG80, Vreden, Germany) and was situated in the central hallway of the pig building. A wooden aisle helped pigs to walk individually and forward over the balance (2.5 m × 0.6 m; Fig. 1a; Additional File 2Video S1). Body weights were registered electronically and coupled to the pig’s ID using an RFID-reader and custom-made software. The camera (Dahua IPC-HDW4831EMP-ASE, Dahua Technology Co., Ltd, Hangzhou, China) was mounted 2.5 m above floor at the center of the weighing scale. Pigs were recorded from an overhead camera perspective with a frame rate of 15 frames per second and a resolution of 3840 × 2160. An example of our data collection and a video recording is provided in Fig. 1b.

### Body part tracking using DeepLabCut

DeepLabCut 2.2b.8^27^ was installed in an Anaconda environment with Python 3.7.7.^30^ on a custom-built computer running a Windows 10 64-bit operating system with Intel Core i5-vPro CPU processor (2.60 GHz) and 8 GB RAM memory. Training, evaluation and analysis of the neural network was performed using DeepLabCut in the Google Colaboratory (COLAB) (https://colab.research.google.com/).

**Figure 2:**
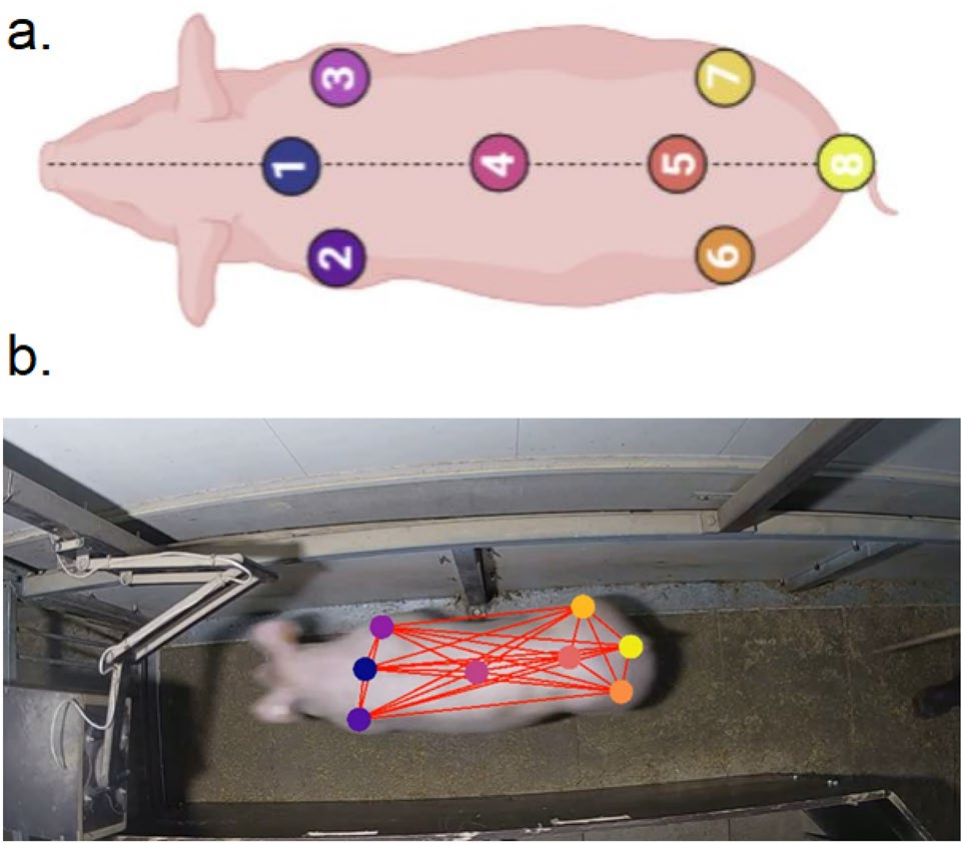
(**a**) Schematic overview of the eight body positions annotated for pose configuration in DeepLabCut^27^ (created with BioRender.com). 1 = Spine1; 2 = Shoulder left; 3 = shoulder right; 4 = Center; 5 = Spine2; 6 = Hip left; 7 = Hip right; 8 = Tail base. (**b**) Example of a labeled pig during weighing using the DeepLabCut software.

To detect body parts on a pig that is walking through the experimental setup, a neural network was trained using DeepLabCut 2.2b^27^ as described in Nath et al.^32^. A minimalistic eight body part configuration (Fig. 2a; Table 1) was necessary to estimate hip width, shoulder width and body length. Operational definitions can be found in Table 1. Head body parts (Nose, Ear left, and Ear right) were also labeled, but not included in our final structural model as these body parts were frequently occluded in consecutive frames.

**Table 1:** Operational definitions of pigs’ body parts during labeling.

Term	Operational definition
Spine1	Spinal location where the shoulders are articulated to the spine
Shoulder left	Caudal part of major tubercle left
Shoulder right	Caudal part of major tubercle right
Center	Spinal location underneath the rib cage. This spinal location functions as a hinge as the pig changes its direction of movement
Spine2	Tuber sacrale: spinal location where the hips are articulated to the spine
Hip left	Caudal part of major trochanter left
Hip right	Caudal part of major trochanter right
Tail base	Base of the tail
Body length	The distance between Spine1 and the tail base
Hip width	The distance between hip left and hip right
Shoulder width	The distance between shoulder left and shoulder right
Surface area	The area of the polygon formed by the body part positions of hip left, shoulder left, spine 1, shoulder right, hip right and tail base

Seven videos of approximately one hour recorded on two different days were selected to include variable pig sizes (20–120 kg) and each video contained multiple pig weighings. From these seven videos, several frames were extracted for annotation using k-means clustering in DeepLabCut. We first annotated 457 frames (~ 1 frame per pig) which were split into a training dataset (95%; 434 frames) and a test dataset (5%; 23 frames). The network was trained in Google Colaboratory using the ResNet-50 architecture with a batch size of 2. We trained our algorithm until the loss function reached an optimum, which indicated a minimal loss with a minimum number of iterations in this study. Next, we compared mean pixel errors of several models within this optimal region. Models with lowest mean pixel errors were visually checked for body part tracking performance on entire videos. Hereafter, the model that performed optimal was tested for flexibility using unseen single pig videos with pigs of variable size (20 vs 120 kg) weighed on different days. As model performance was suboptimal at first, poorly tracked outlier frames were extracted using the DeepLabCut ‘jump’ algorithm^32^. This algorithm identifies frames in which one or more body parts jumped more than a criterion value (in pixels) from the last frame^32^. These outlier frames were refined manually and hereafter added to the training dataset for re-training. In total, 150 outlier frames were extracted from six novel videos containing one single pig to improve tracking performance (± 25 frames per pig). The final training dataset consisted of 577 (95%) frames and a test dataset of 30 frames (5%). The network was then trained again using the same features as the first training. Additional File 3Video S2 shows an example of a pig with body part tracking.

### Extracting weight subsets and body dimension estimation

After posture extractions of body parts using DeepLabCut, body dimension parameters were estimated. The raw dataset contained body part positions and tracking probabilities of 5,102,260 frames. Individual pig IDs were first coupled with video recordings based on time of measurement from the weight dataset. The following steps and analyses were performed in R^33^. Frames with a mean tracking probability < 0.1 over all eight body parts were removed (2,792,252 frames left). This large reduction in number of frames (± 50% removed) was mainly caused by video frames without any pigs, for example in between weighing of different pens or in between weighings of pigs.

**Figure 3:**
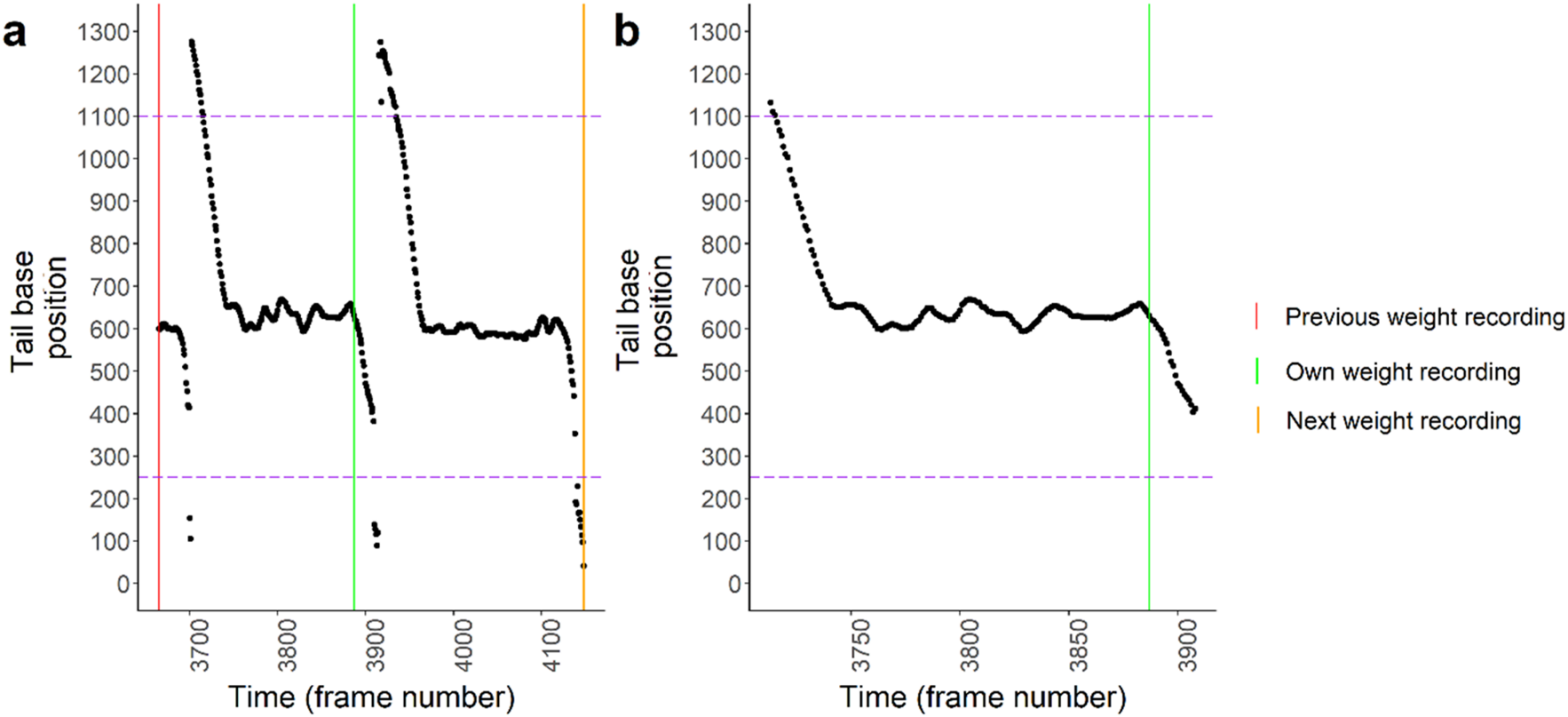
Determination of time window for a weight recording. (**a**) First, a subset is created as all tail base x-positions between time of recording of the next (orange) and previous (red) weight recording. The start time of the time window is determined as the first value before the own weight recording (green) above the threshold of 1100 pixels (dashed purple line; pig entering weighing scale). The end time of the time window is determined as the first value after the own weight recording (green) below the threshold of 250 pixels (dashed purple line; pig leaving weighing scale). (**b**) The extracted time window on which body part dimensions will be estimated and trajectory analysis will be performed.

Next, for every weighing event, start and end points were determined to estimate body dimensions and activity traits. For a specific weighing event, a subset was first created containing all frames between the previous and next weighing event. The time of entrance and departure of the pig on the weighing scale was estimated using the x-position (in pixels) of the tail base, as the movement of pigs was predominantly along the x-axis (from right to left; Fig. 2b). The frame of entrance was defined as the first frame of a subset where the rolling median (per 10 frames) of the tail base x-position exceeded 1100 pixels (Fig. 3). Likewise, the first frame after a pigs’ weighing event with a rolling median tail base x-position < 250 pixels was used to determine time of departure. If these criteria were not met, the first frame and/or the frame at which the weight record took place were used for the time of entrance/departure.

Hip width, shoulder width and body length of a pig were estimated by using the median value of the distance between certain body parts over all frames for a specific weight recording (Table 1, Fig. 2). These body dimensions in pixels, were transformed to metrics as 1 cm was calculated to be equivalent to 29.1 pixels. The conversion ratio from pixels to centimeters was based on the distance between tiles of the weighing scale, which was known to be exactly 50 cm. Total surface area was estimated using the mean value of the area calculated with the *st_area* function in R from the R-package *sf*^34^ using all outer body part locations. Standard deviations of the body part positions were also calculated for all frames between entrance and departure after quality control (as described above), to assess the stability of estimates.

### Estimation and interpretation of activity traits

Trajectory analysis was performed using the R-package ‘trajr’^35^ for left and right shoulder, left and right hip and the tail base. For each body part, pixel coordinates were extracted, trajectories were rescaled from pixels to cm and a smoothed trajectory was created using the *TrajSmoothSG* function. From these smoothed trajectories, the following activity-related features were derived: mean and standard deviation of speed and acceleration (‘TrajDerivatives’), a straightness index (‘TrajStraightness’) and sinuosity (‘TrajSinuosity2’).

The straightness index and sinuosity are related to the concept of tortuosity and associated with an animals’ orientation and searching behavior^35,36^. The straightness index is calculated as the Euclidean distance between the start and the endpoint divided by the total length of the movement^36^. The straightness index is an indication of how close the animal’s path was to a straight line connecting the start and final point and varies from 0 to 1. Thus it quantifies path efficiency whereas the closer to 1, the higher the efficiency. In our experiment, this path efficiency will be highest when a pigs walks in a straight line during weighing (straightness index = 1). Any deviations from this straight line—due to an increased activity of the pig during weighing—will lower the straightness index towards zero. Sinuosity tries to estimate the tortuosity of a random research path by combining step length and the mean cosine of an animals’ turning angles^35–37^. The sinuosity of a trajectory varies between 0 (random movement) and 1 (directed movement).

In this study we hypothesize that mean speed, straightness index and sinuosity are related to pigs’ activity during weighing. In an extreme case, a pig will walk in a straight line towards the RFID reader, stand motionless until weight is recorded and continues its walk in a straight line after the gate is opened. This would result in a low mean speed (m/s), a sinuosity > 0 and a straightness index of 1. We hypothesize that more active pigs will present more lateral movements, increasing the mean speed and lowering the straightness index and sinuosity. So generally, more calm pigs during weighing will display a lower mean speed, although they might have run with a high speed towards the RFID reader.

### Validation of body dimension estimation and activity traits

The estimations of body dimensions using video recordings analyzed with DeepLabCut were validated by an independent set of 60 pigs after the initial experiment. These pigs came from five pens of different ages (92–166 days) and were measured manually for tail-neck length and hip width using a simple measuring tape. Pig surface area was estimated for the manual recordings as the multiplication of tail-neck length and hip width. The manual estimates for tail-neck length, hip width and pig surface area were then compared to the estimates from the video analysis by calculating Pearson correlations and root mean squared error (RMSE).

Automated activity traits were validated by comparing these values with manual activity scores given by five trained observers. Video footage of 1748 pig weighings were manually scored for pig activity by at least two observers per pig on a scale from 1 (calm) to 5 (very active). This ordinal activity scale was constructed based on D’Eath et al. and Holl et al.^17,24^. The average activity score per pig was then compared with automated activity scores by calculating Pearson correlations.

### Quality control of estimated variables

After estimation of body dimension and activity traits, additional quality control was performed. First, estimates of hip and shoulder width, tail-neck length and pig surface area were set to missing for records with frame by frame standard deviation estimates higher than the mean + 3 × standard deviations for all records. The thresholds were 10.2 cm for hip distance (132 records), 11.8 cm for shoulder distance (135 records), 20.6 cm for tail-neck length (121 records) and 0.058 m^2^ for pig surface area (96 records). If the standard deviation of the estimated hip widths over frames within one weighing event of a pig was > 8.9 cm, the record was set to missing.

Second, for every individual with at least four records (941 pigs, 6807 records), outliers were determined using a second order polynomial regression on the variable of interest in function of age in days. Based on the distribution of the difference between observed and predicted phenotypes for all animals, a threshold for exclusion (record set to missing) was set as three times the standard deviation of the differences. The thresholds were 2.1 cm for hip distance (61 records), 2.2 cm for shoulder distance (58 records), 6.4 cm for tail-neck length (75 records), 0.021 m^2^ for pig surface area (85 records) and 3.7 kg for weight (86 records).

The final dataset after data cleaning included 7428 records from 1556 finishing pigs descending from 73 Piétrain sires and 204 crossbred dams. Pedigree comprised 4089 animals, where the median pedigree depth of Piétrain sires was 15 generations (min 10; max 17) and 3 (min 0; max 6) for crossbred dams.

### Genetic modelling

We estimated genetic parameters (heritability and genetic correlations) using the *blupf90* suite of programs^38^. Genetic variances and heritabilities were estimated with average information REML, implemented in *airemlf90* and invoked with the R-package *breedR*^39^ with the options “EM-REML 20”, "use_yams" and “se_covar_function”. Genetic parameters were first estimated on the full dataset and thereafter on subsets per pigs’ weight recording (1 to 8). The first weight recording, for example, corresponds with a dataset of 1176 pigs between 78 and 89 days of age (Table 2). We estimated h^2^ as the proportion of additive genetic variance divided by total variance, whereas the common environmental effect (c^2^) was estimated as the proportion of variance explained by random environmental effects (c)*,* divided by total variance.

**Table 2:** DeepLabCut pose estimation prediction errors (in pixels) with or without probability cut-off (p-cut-off) values compared to the ground truth (manual annotations).

p-cut-off	Train error in pixels (and in cm)		Test error in pixels (and in cm)	
	None	0.1	None	0.1
All	99.2 (3.4)	81.1 (2.8)	95.9 (3.3)	76.1 (2.6)
Spine1	87.8 (3.0)	80.6 (2.8)	90.0 (3.1)	87.4 (3.0)
Shoulder left	154.8 (5.3)	136.0 (4.7)	102.3 (3.5)	99.8 (3.4)
Shoulder right	125.3 (4.3)	107.2 (3.7)	119.5 (4.1)	127.7 (4.4)
Center	94.6 (3.3)	78.6 (2.7)	89.7 (3.1)	68.5 (2.4)
Spine2	89.0 (3.1)	36.3 (1.2)	108.7 (3.7)	14.0 (0.5)
Hip left	97.3 (3.3)	86.7 (3.0)	99.8 (3.4)	101.5 (3.5)
Hip right	88.2 (3.0)	83.0 (2.9)	48.8 (1.7)	48.8 (1.7)
Tail base	56.3 (1.9)	37.8 (1.3)	107.7 (3.7)	51.6 (1.8)

For every DeepLabCut prediction, a likelihood is calculated and a p-cut-off can be defined to filter unreliable predictions. One cm was estimated to be equivalent to 29.1 pixels.

Genetic correlations (r_g_) between traits were estimated using bivariate animal models (*airemlf90*). Genetic correlations were first calculated between all possible trait combinations using the full dataset. Hereafter, the genetic correlations within traits for all pairwise weighing events were estimated (so two recordings of the same trait were treated as two different traits). By doing this, we can evaluate if a trait genetically changes over time.

The estimated animal models were of the form:

$$y=Xb+Za+Wc+e$$

where *y* is the vector with phenotypes for the studied trait(s); *b* is the vector containing the fixed effects (sex, 2 levels; parity of dam, 4 levels) and covariates (age); *a* is the vector of additive genetic effects (4089 levels); *c* is the vector of random environmental effects (65 levels); *e* is the vector of residual effects; *X*, *Z* and *W* are incidence matrices for respectively fixed effects, random animal effects and random permanent environmental effects. The random environmental effect *c* is a combination of date of entrance at the fattening farm and weighing date. Every two weeks, a new batch of pigs arrived at fattening farm. Parity of dams consisted of four classes (‘1’, ’2–3’, ‘4–5’, ‘6 +’)’.

## Results

### Performance of body part tracking using DeepLabCut and validation

Performance of the network was evaluated by computing both train and test errors. These errors are measured by the average Euclidian difference between the pixel coordinates from the manual annotations and the DeepLabCut estimations on the training dataset and test dataset. The mean pixel error on the training dataset without probability cut-off (p-cut-off) was 99.2 pixels or 3.4 cm and 95.9 pixels or 3.3 cm for the test dataset (Table 2) for the final network (Additional File 3Video S2). Applying a p-cut-off (p = 0.10) improved mean pixel error on the training dataset down to 81.1 pixels or 2.8 cm and 76.1 pixels or 2.6 cm for the test dataset. After p-cut-off, mean pixel errors of individual body parts ranged between 14.0 and 136.0 pixels equivalent to 0.5 and 4.7 cm.

To validate the performance of the automated pose estimation algorithm, estimated tail-neck length, hip width and pig surface area were compared with manual recordings. Pearson correlations between manual recordings and video analysis were high for tail-neck length (r = 0.94; RMSE = 3.2 cm), hip width (r = 0.80; RMSE = 1.8 cm) and pig surface area (r = 0.91; RMSE = 0.019 m^2^). However, estimates using video analysis were on average 7.4 cm higher for tail-neck length and 2.3 cm for hip width. Moreover, automated activity scores were validated by comparing them with manually obtained activity scores given by trained observers. Pearson correlations between manual recordings and video analysis were moderate to high for mean speed (r = 0.49), straightness index (r = − 0.57) and sinuosity index (r = − 0.32). After combining these three automated activity traits in an ‘activity index’ (1/3 weight per trait after rescaling), Pearson correlation with manual activity scores increased to r = 0.62. The inter-observer Pearson correlation for activity score was moderate to high as well, ranging from r = 0.55–0.84. Pairwise correlation plots for all validations are provided in Additional File 4 Figure S1.

### Descriptive statistics

**Figure 4:**
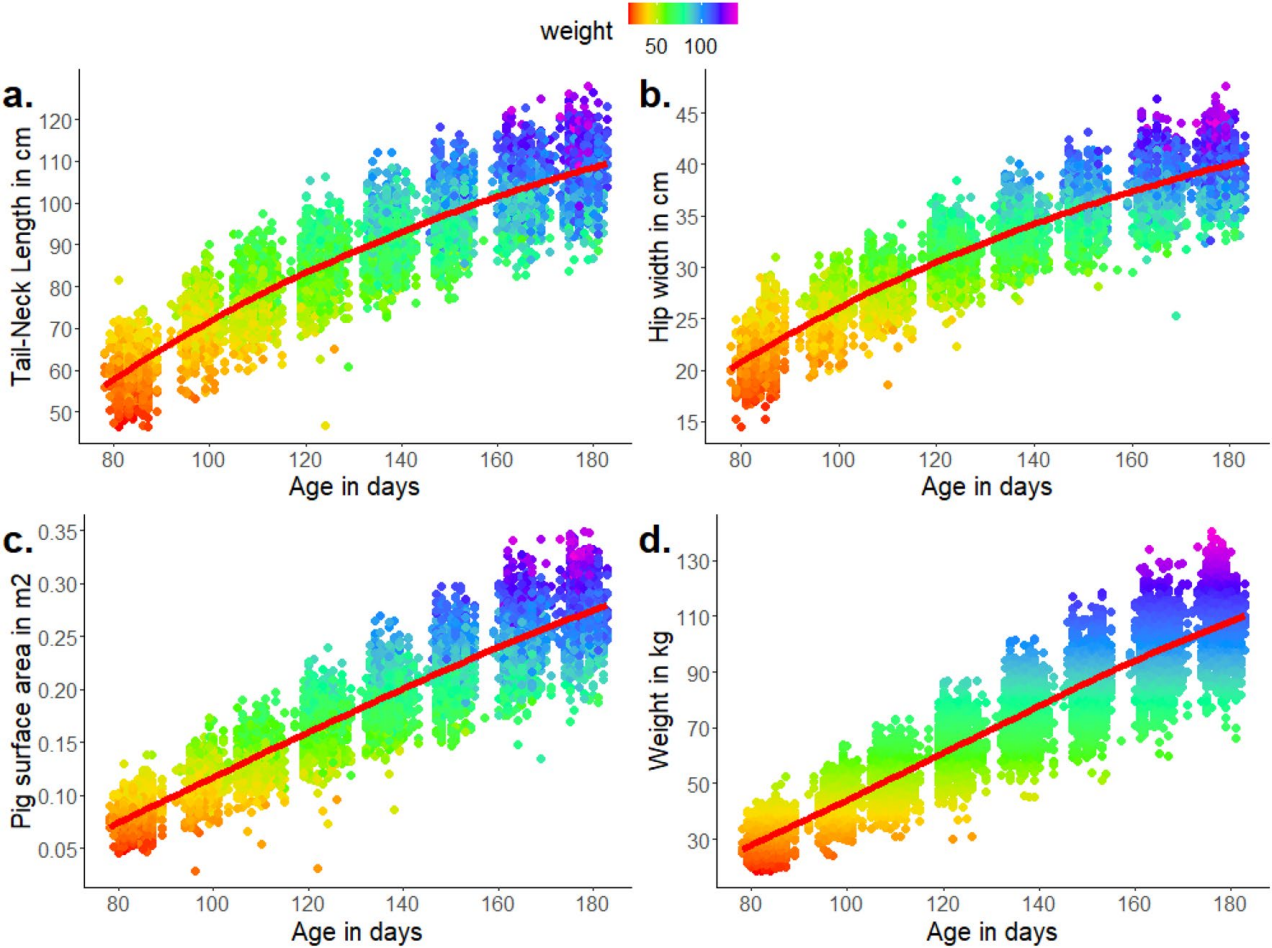
Evolution of body dimensions in function of age (in days) and colored by weight (in kg). A smoothed regression line is shown in red. Per pig, a maximum of eight recordings took place every fourteen days, explaining the eight separate data ‘blocks’ with whitespaces in between. (**a**) Evolution of tail-neck distance. (**b**) Evolution of hip width. (**c**) Evolution of pig surface area. (**d**) Evolution of weight. The graph for shoulder width is not shown, as it was equivalent to hip width.

An overview of descriptive statistics of the most important traits is shown in Table 3. The traits were approximately normally distributed based on visual inspection of histograms. Figure 4 shows the evolution in body dimensions and weight as a function of pigs’ age. The steepness of the growth in tail-neck length, hip width and shoulder width decreases over time, in contrast to pigs’ weight and surface area, which show an approximately linear increase.

**Table 3:** Descriptive statistics of most important traits for the first recording, thirteen days after arrival of pigs in the fattening farm (N = 1176) and for the last (eighth) recording (N = 743).

Trait	First weight recording		Eighth weight recording	
	Mean (sd)	Range	Mean (sd)	Range
Age (days)	83.4 (2.2)	78.0–89.0	175.9 (3.9)	162.0–182.0
Weight (kg)	30.6 (5.1)	18.5–52.5	106.2 (13.0)	66.0–140.5
Average daily gain (g/day)	367 (59)	220–603	604 (74)	365–798
Hip width (cm)	21.7 (2.4)	14.5–31.0	39.7 (2.6)	25.3–47.6
Shoulder width (cm)	20.5 (2.1)	15.7–30.0	37.8 (2.9)	28.4–46.0
Tail-neck length (cm)	60.1 (4.8)	44.8–81.5	107.1 (7.5)	86.3–127.9
Pig surface area (m^2^)	0.081 (0.014)	0.044–0.137	0.268 (0.032)	0.135–0.349
Meat percentage (%)	–	–	63.4 (3.1)	51.9–70.3
Feed intake (kg/day)	–	–	1.92 (0.16)	1.60–2.45
FCR (kg/kg)	–	–	2.48 (0.10)	2.27–2.78
Duration of weighing (s)	20.5 (11.3)	6.8–59.6	18.5 (7.7)	3.1–55.4
Mean speed (m/s)	1.2 (0.4)	0.3–3.9	1.1 (0.4)	0.3–3.7
SD speed (m/s)	1.6 (0.7)	0.2–6.4	1.3 (0.5)	0.5–4.0
Absolute acceleration (m/s^2^)	11.6 (7.5)	2.1–72.3	9.1 (7.0)	2.2–84.4
SD acceleration	23.4 (16.8)	3.4–124.7	16.5 (16.2)	3.3–165.3
Straightness index	0.488 (0.190)	0.017–0.928	0.489 (0.171)	0.071–0.918
Sinuosity index	0.296 (0.056)	0.168–0.620	0.299 (0.063)	0.140–0.547

Activity traits shown are derived from the tail base. Note that the traits meat percentage, feed intake (pen level) and feed conversion ratio (pen level) were recorded after slaughter of pigs and do not completely correspond with the last video recording. The fastest growing pigs were slaughtered a few days after the last video recording, whereas this took more than 30 days for the slowest growing pigs. *FCR* Feed Conversion Ratio.

### Repeatability of traits

Repeatability of traits was assessed by looking at the phenotypical Pearson correlation matrix within a trait over time for the same pigs (Additional File 5 Fig. S2). For body dimensions and average daily gain, repeatability was generally very high and significantly (p < 0.001) larger than zero (r = 0.41–0.98). Lowest correlations were found between first and last recordings. Repeatability was low for the activity traits mean speed, straightness and sinuosity (r = 0.05–0.39), although consistently positive and significantly different from zero for most comparisons (p < 0.001; denoted with *** in Additional File 5 Fig. S2). Moreover, successive recordings (two-week intervals) for activity traits showed a consistent and significant (p < 0.001) Pearson correlation in the order of magnitude of r = 0.3. Furthermore, internal correlations of the order in which pigs have been weighed steadily increased over time from about r = 0.10 to r = 0.40.

### Genetic parameters

Estimates of h^2^ and c^2^ for the full dataset are shown in Table 4, whereas these estimates for subsets per weight recording is given in Additional File 6 Fig. S3. Heritability estimates were high for the estimated body dimension parameters hip width (64.1%), shoulder width (66.4%), tail-neck length (71.9%) and pig surface area (74.8%). For the behavioral parameters, h^2^ was very low for weighing duration (2.9%), standard deviation of tail base speed (6.3%) and mean and standard deviation of tail base acceleration (8.7% and 5.5% respectively). However, h^2^ estimates were low to moderate for mean speed (24.5%), straightness index (25.9%) and sinuosity (23.8%) of tail base and estimates were even higher for these traits computed from left and right shoulder and hip trajectories (22–35%; Additional File 7 Table S1).

**Table 4:** Estimates of heritability (h^2^) and common environmental effects (c^2^) expressed as percentage, as well as additive genetic standard deviation (σ_a_), random permanent environmental standard deviation (σ_c_) and residual standard deviation (σ_e_).

Trait	h^2^ (se)	c^2^ (se)	σ_a_	σ_c_	σ_e_
Average daily gain (g/day)	82.5 (1.4)	7.7 (1.4)	65	20	22
Hip width (cm)	64.1 (2.6)	17.6 (3.1)	2.0	1.0	1.1
Shoulder width (cm)	66.4 (1.8)	10.8 (1.9)	2.0	0.8	1.2
Tail-neck length (cm)	71.9 (1.5)	7.7 (1.4)	5.6	1.8	3.0
Pig surface area (m^2^)	74.8 (0.8)	2.9 (0.1)	0.020	0.004	0.011
Meat percentage (%)	69.8 (8.2)	4.3 (2.8)	2.2	0.5	1.3
Duration of weighing (s)	2.9 (0.8)	2.5 (0.6)	1.4	1.3	7.9
Mean speed (m/s)	24.5 (1.6)	2.2 (0.6)	20.1	6.1	34.7
SD speed (m/s)	6.3 (1.0)	6.6 (1.3)	13.3	13.6	49.4
Mean absolute acceleration (m/s^2^)	8.7 (1.2)	3.8 (0.9)	208	138	661
SD acceleration (m/s^2^)	5.5 (1.0)	5.1 (1.1)	358	347	1449
Straightness index	25.9 (1.6)	0.8 (0.3)	0.09	0.02	0.15
Sinuosity index	23.8 (1.6)	1.7 (0.5)	0.03	0.01	0.05

Activity scores shown are based on estimates from the tail base; All other estimates are shown in Additional File 7 Table S1 Additional File 6 Fig. S3.

**Figure 5:**
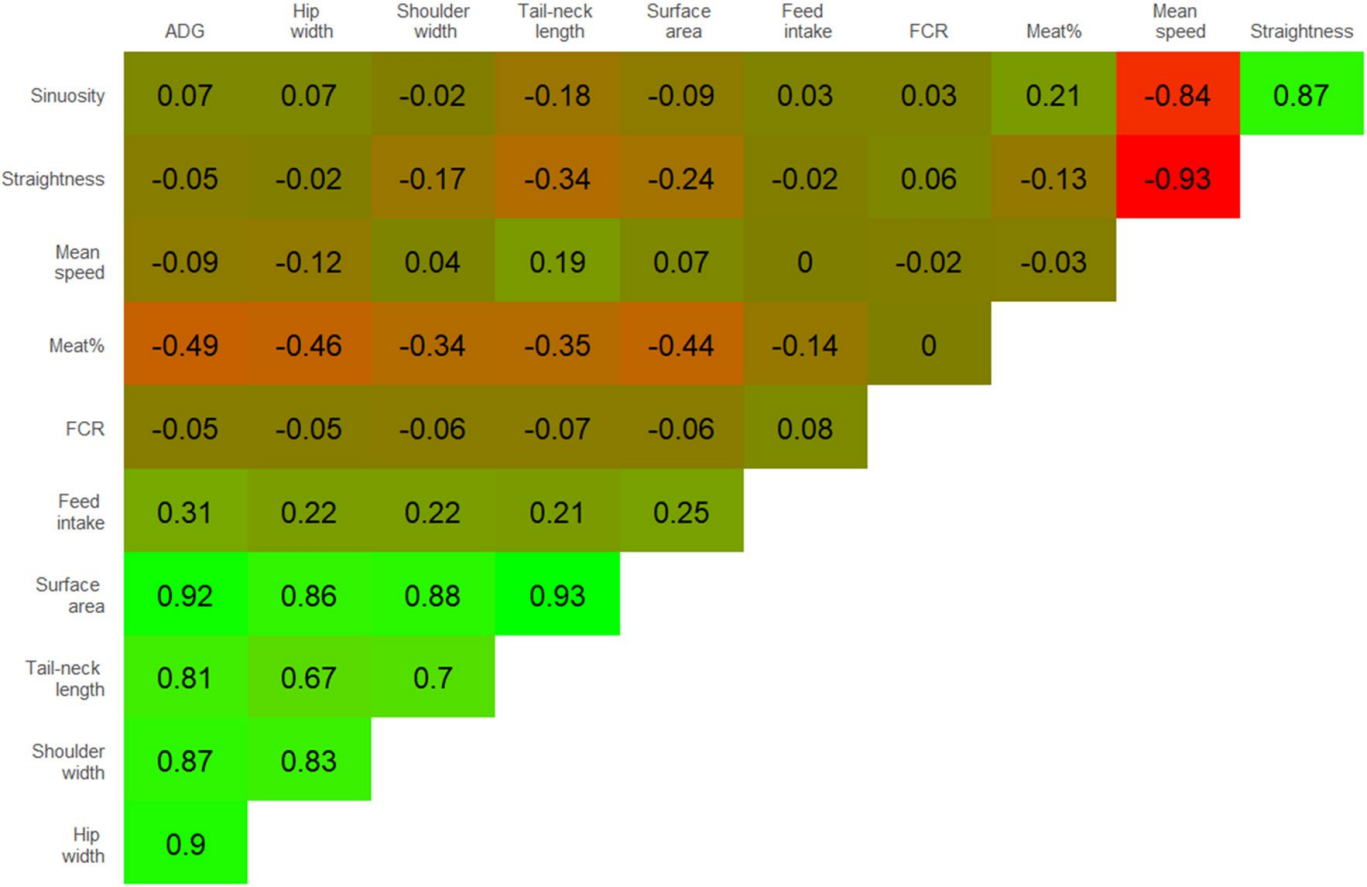
Heatmap of genetic correlation (r_g_) estimates between a set of selected traits for the full dataset. All estimated genetic correlations (with standard errors) can be found in Additional File 8 Table S2.

A selection of the most relevant r_g_ estimates for the full dataset are shown in Fig. 5, a list with all bivariate genetic correlations for all possible trait combinations is given in Additional File 8 Table S2. High genetic correlations (r_g_ = 0.81–0.92) were observed between body dimension parameters and ADG, and low to moderate negative correlations (r_g_ = − 0.46 to − 0.34) were found between body dimension parameters and meat percentage. Mean speed was highly negatively correlated with straightness index (r_g_ = − 0.93) and sinuosity (r_g_ = − 0.84). Low genetic correlations (r_g_ = − 0.34–0.19) were observed between body dimension parameters and behavioral parameters. Very low genetic correlations were estimated between behavioral parameters and tail biting, ear biting and ear swellings (Additional File 8 Table S2).

## Discussion

Behavioral analysis is becoming central in the assessment of animal welfare, a keystone of modern, sustainable pig breeding. The overall goal of this study was to expand routine pig weighing procedures to include automated measurements of body composition and activity levels. Using DeepLabCut (DLC) software, we developed a model for pose estimation of individual fattening pigs. We were able to estimate relevant body parts accurately with an average tracking error of 3.3 cm. Using the tracking output we were able to estimate body dimensions and behavioral activity traits. DLC estimations were validated by using manually collected body dimensions as a golden standard. Pearson correlations between the automated estimations and manual observations were high, ranging between 0.80 and 0.94. Moreover, automated activity scores had moderate to high correlations with manually scored activity traits (r = 0.32–0.62). This validation indicates that our methodology is adequate in quantifying general pig activity, certainly since inter-observer correlations for activity were similar (r = 0.55–0.84).

Focusing only on increased production comes at the cost of an increased amount of production-related diseases and disorders such as leg problems in fattening pigs^40^. Combining genetics with analysis of body conformation and animal behavior may help designing more sustainable and robust breeding in the future^40^. However, defining and scoring behavioral read-outs in pigs on a large scale for application in breeding programs remains problematic. Additionally, large datasets with a sufficient pedigree structure are required to estimate heritabilities and genetic correlations accurately^41^. Here, we were able to combine genetic information with direct behavioral read-outs. Heritabilities of body dimension parameters were high (h^2^ = 61–74%) and even somewhat higher than estimates found in literature (h^2^ = 30–60%)^14,15^. This could be attributed to high standardization and low environmental variability in our study: all measurements took place in the same experimental fattening farm within the same season (January–July 2021). This is also reflected in the low estimates for permanent environmental effects (c^2^ = 7.7–17.6%) for these traits. Heritability estimates were low to moderate for the activity traits mean speed, straightness index and sinuosity (h^2^ = 22–35%). These estimates are similar to those of manually scored activity and handling traits in pigs during weighing (h^2^ = 10–23%)^3,17–19,24^.

No adverse correlations were found between activity traits and body dimension parameters (r_g_ = − 0.34–0.19), which indicates that pigs can be selected for both types of traits simultaneously. These findings are in line with Holl et al.^17^ reporting low genetic correlation between activity score and back fat thickness (r_g_ = − 0.11 to − 0.16) as well as a moderate association between activity score and growth (r_g_ = − 0.38). Similar to Ohnishi and Satoh^42^, genetic correlations were high between body dimension parameters and average daily gain (r_g_ = 0.81–0.92). Despite these high genetic correlations, combining body dimension parameters with weight recordings remains relevant in (pig) breeding. Body length, for example, is correlated with the number of vertebrae, teats and litter size^43,44^. Activity traits mean speed, straightness index and sinuosity were highly correlated, presumably because these traits can be traced back to the activity level of pigs during weighing.

Low correlations were found between tail or ear biting scores, body dimension and activity parameters. Hence, reducing tail biting as a correlated response from selecting pigs based on these activity traits does not seem feasible. Ursinus et al.^45^ also reported that tail biting is difficult to predict by individual behavior. There are indications, however, of a low to moderate relationship between activity during weighing and aggression in the pen^3,24^. Therefore, breeding pigs with reduced activity during weighing might lower aggression and injuries. Selective breeding against undesired behaviors, such as aggression, has already shown to be effective in pigs^46^.

Unfortunately, we were unable to compare our activity traits during weighing to activity in the pen. However, studies have found a relationship between activity during weighing and aggression in the pen^3,24^, whereas others linked changes in activity scores in the pen to tail biting and infections^16,20–22^ or residual feed intake^23^. However, currently evidence is lacking on the relationship between activity during weighing and activity in the pen. This relationship between weighing activity and pen activity needs further research. It should be noted that, in our setting, a single pen (typically 13 pigs) was first brought to a central hallway, after which pigs were weighed one after the other. Pigs usually reached the RFID reader within a few seconds, after which it took about 10–20 seconds to actually weigh them. Hence, the mean speed we estimate mainly indicates to which extent a pig moved back and forth while being weighed. Moreover, sinuosity and straightness indices are mostly used in ecological studies under the assumption that animals can move freely^35,36^. In our setup, pigs were restricted to a limited area of approximately 2.5 m × 0.6 m (Fig. 1). Although interpretation is different, we argue that these traits are still very relevant to characterize pig behavior, since they provide a good indication of activity during weighing.

Although our neural network was developed for specific characteristics and settings (i.e., white finishing pigs, weighed individually on a large weighing scale), the network can be relatively easily generalized or expanded, as explained by Winters et al.^47^. A practical limitation of our procedure was that videos were analyzed after recording and had to be stored, and afterwards coupled with the weight dataset (containing IDs). This limitation could be tackled by using a real-time version of DeepLabCut in combination with a RFID reader^48^ or improvements in animal identification using computer vision systems, which currently is a major challenge. The developed model could also be expanded to detect very specific pig behaviors during weighing, such as jumping or turning around (U-turns), using SimBA (Simple Behavioral Analysis) software (https://github.com/sgoldenlab/simba)^49^. During our experiment, several pigs tried to escape the weighing scale by trying to jump out, which might be an indication of flighty animals^3^. Furthermore, our model could be expanded to identify tail and ear biting marks and/or skin injuries, as demonstrated by Blömke et al.^50^. Adding a side view camera and/or 3D-camera would possibly allow us to refine our analyses, although it would increase complexity, and therefore limit its on farm use in large scale programs. This would enable us to estimate pig height, muscle depth and back fat^5^, or perform gait analysis related to lameness or other locomotor problems^51^.

## Conclusions

In the present study, we estimated pigs’ body dimension and activity traits using automated pose estimation on recorded videos. Our methodology expands the standard routine of pig weighing with a computer vision system, which is able to accurately phenotype both pigs’ body dimensions and activity traits. Moreover, we validated our results and showed these traits are heritable and show no adverse genetic correlation with production traits. These methods are valuable for (pig) breeding organizations to phenotype new production and behavioral traits automatically.

## Supplementary Information

Supplementary Information 1.

Supplementary Video 1.

Supplementary Video 2.

## Data Availability

The dataset will be made accessible upon motivated request. All our annotated images and tracking models are available on: 10.17605/OSF.IO/QKW5Y. For further inquiries, please contact the corresponding author.

## Abbreviations

ADG: Average daily gain
c^2^: Common environmental effects
CVS: Computer vision systems
DLC: DeepLabCut
FCR: Feed conversion ratio
h^2^: Narrow sense heritability
r: Pearson correlation
RFID: Radio frequency identification
r_g_: Genetic correlation
