# Supplementary material for: Estimating genetics of body dimensions and activity levels in pigs using automated pose estimation
Source: Sci Rep. 2022 Sep 13;12:15384. doi: 10.1038/s41598-022-19721-4 (PMC9470733; doi:10.1038/s41598-022-19721-4)
Supplement: Supplementary file 1 — Supplementary Information 1. [file 41598_2022_19721_MOESM1_ESM.docx]

**Supplementary Files**

# **Additional File 1. Score sheet for observed physical abnormalities**

**Each pig was scored manually during weighing on the following physical abnormalities: ear swellings or hematomas (0=none, 1=one ear, 2=both ears); the presence and size of umbilical hernia (0=not present, 1=present); ear biting wounds (0=none, 1=one ear, 2=both ears) and tail biting wounds (0=none, 1=small scratches, 3=bloody and/or infected tail**

|  | Normal | Mild-Moderate | Severe |
| --- | --- | --- | --- |
| Tail biting | “0” | “1” | “3” |
|  | 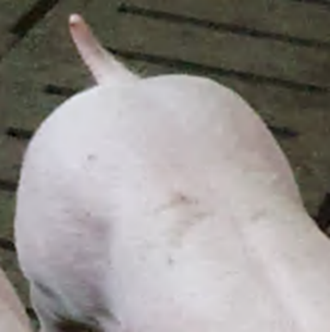 | 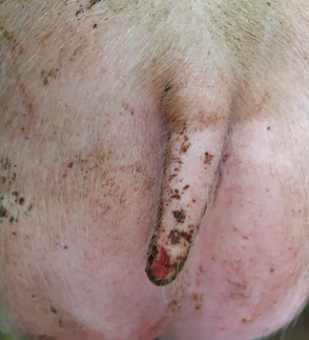 | 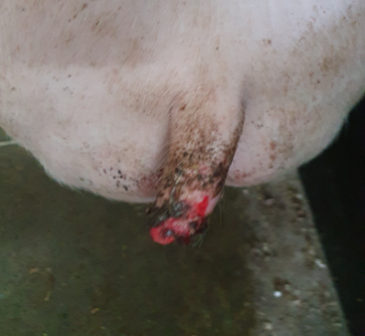 |
| Ear biting | “0” | “1” | “2” |
|  | 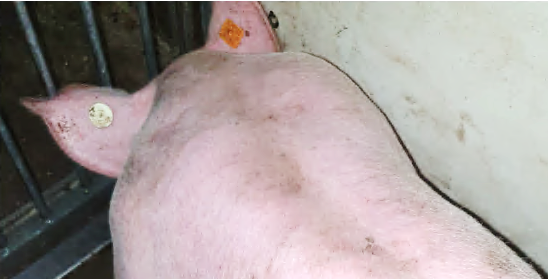 | 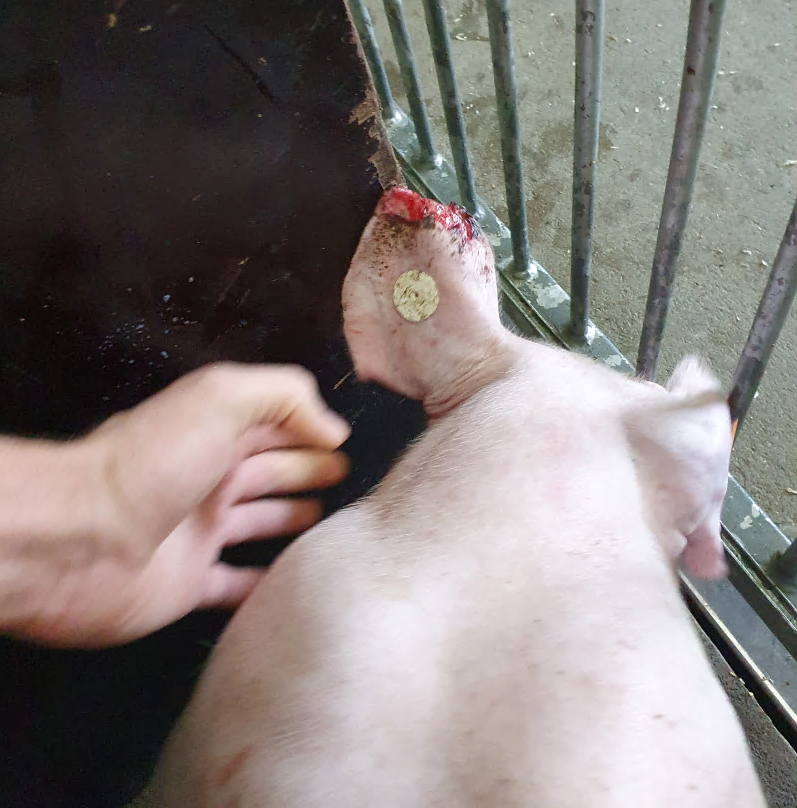 | 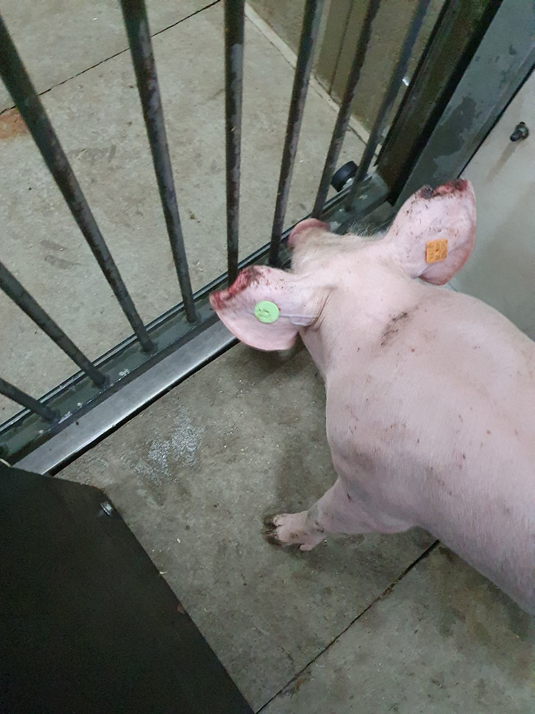 |
| Ear swelling | “0” | “1” | “2” |
|  | 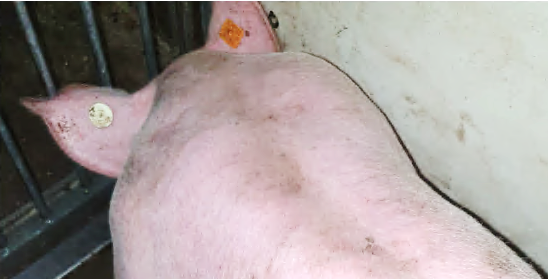 | 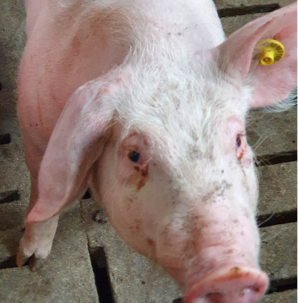 | 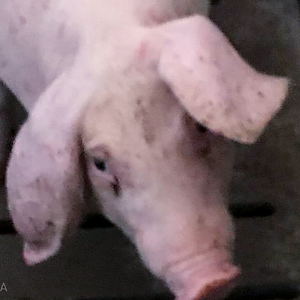 |
| Umbilical hernia | “0” | “1” | - |
|  | 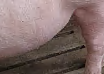 | 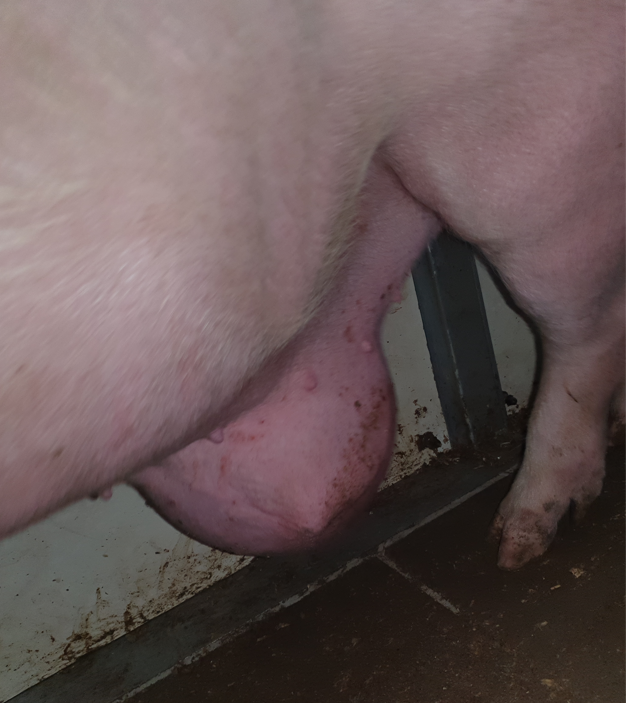 |  |

# **Additional File 2 Video S1. Example of pig weighing procedure**

# **Additional File 3 Video S2. Example of pig with labeled body parts during weighing**

# **Additional File 4 Figure S1. Pairwise correlation plots of validations**

**In the pairwise correlation plots below, Pearson correlations are shown between parameters from automated video analysis and manually obtained records for pigs’ body dimensions and activity levels. Significance values to test if a correlation differs from zero are denoted with ° (p<0.10), * (p<0.05), ** (p<0.01) and *** (p<0.001).**


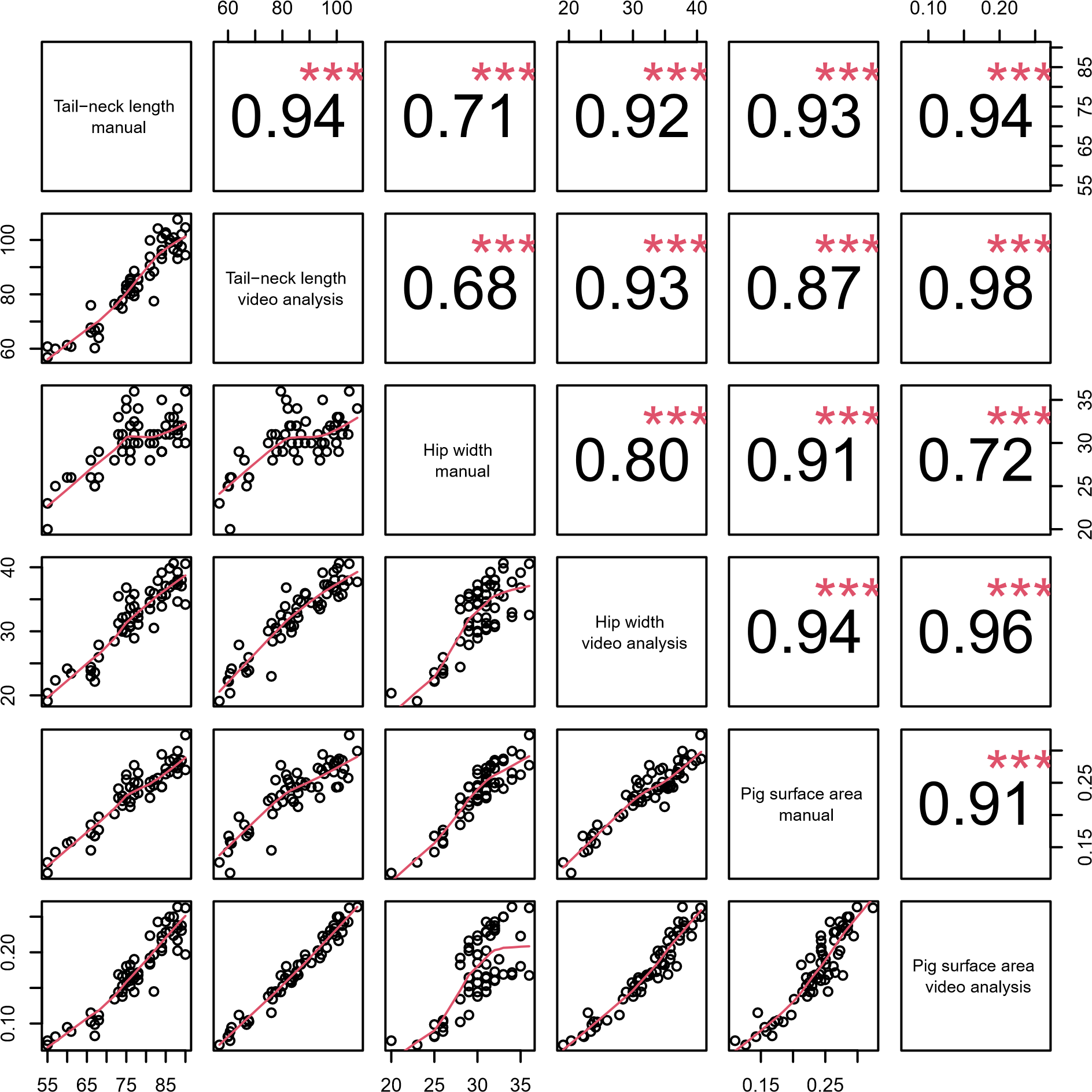


Tail−neck length manual

55

60

65

70

75

80

85

90

60

70

80

90

100

60

70

80

90

100

55

60

65

70

75

80

85

90

0.94

***

Tail−neck length video analysis

Hip width manual

20

25

30

35

20

25

30

35

40

20

25

30

35

40

20

25

30

35

0.80

***

Hip width video analysis

Pig surface area manual

0.15

0.20

0.25

0.30

0.10

0.15

0.20

0.25

0.10

0.15

0.20

0.25

0.15

0.20

0.25

0.30

0.91

***

Pig surface area video analysis


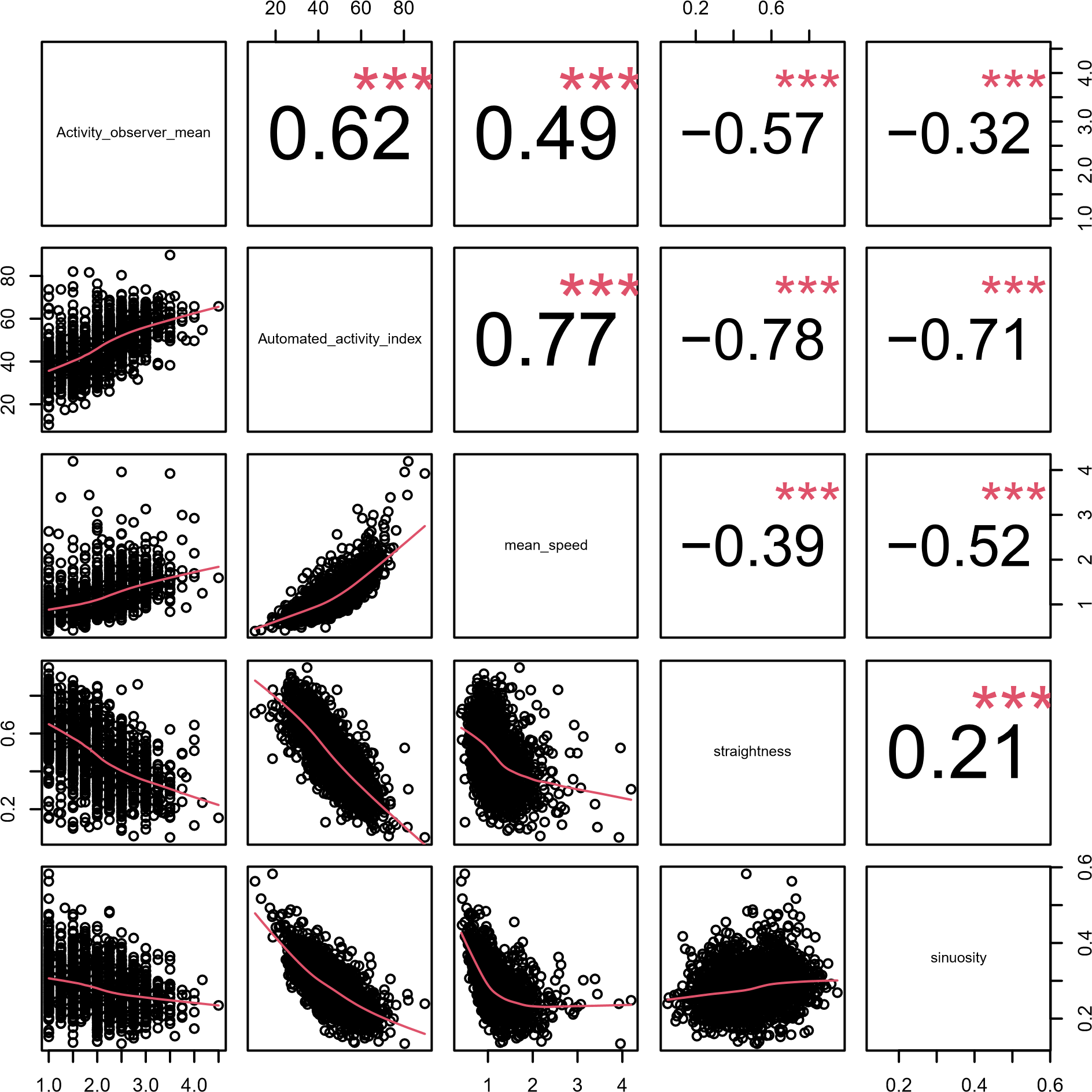


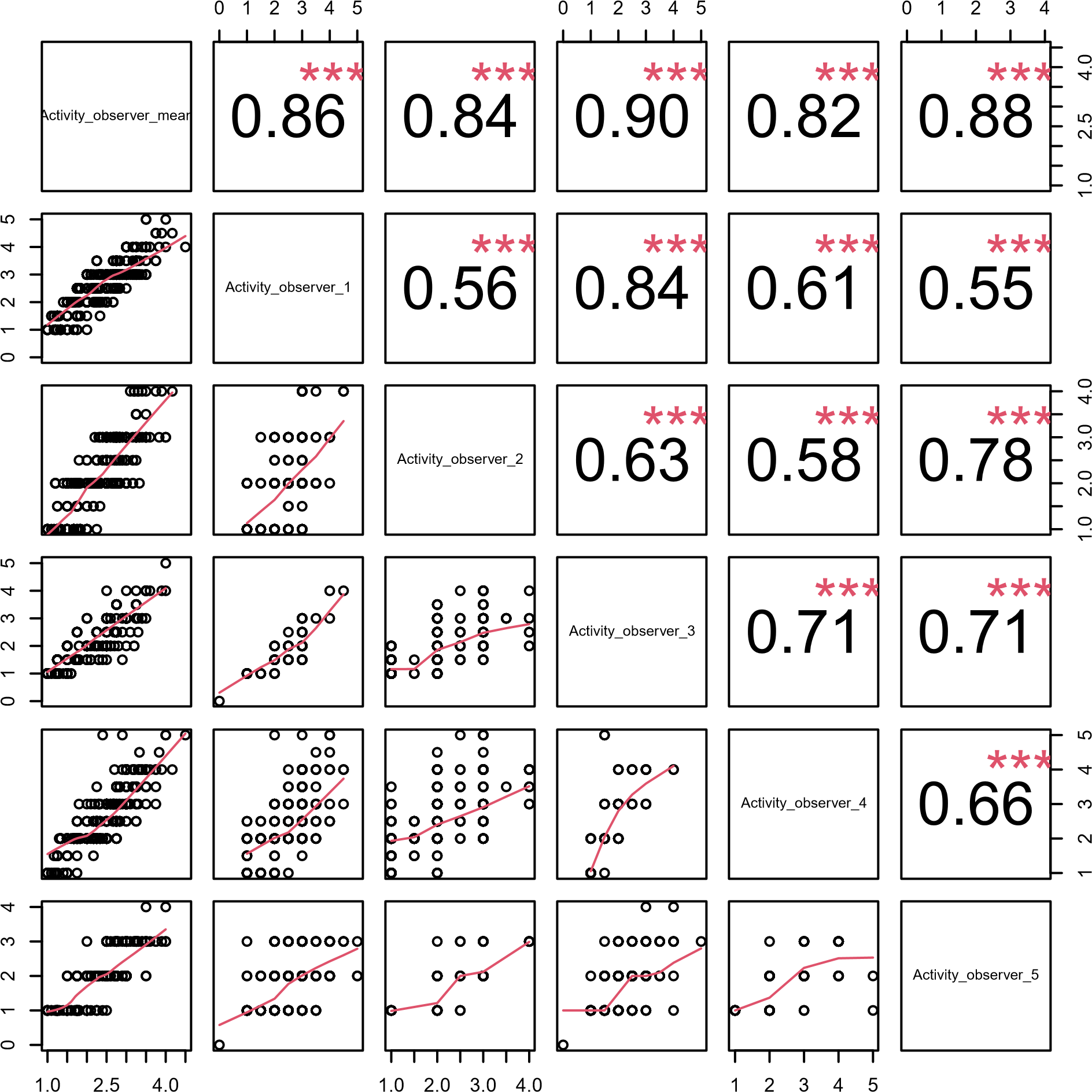


# **Additional File 5 Figure S2. Repeatability of different traits over weighing events**

**In the pairwise correlation plots below, Pearson correlations are shown of repeatedly recorded phenotypes from the same pigs over different weighing events for a range of traits. After each trait name, the event number is given, for example “1” for the first weighing event and “8” for the eighth and last weighing event. Significance values to test if a correlation differs from zero are denoted with ° (p<0.10), * (p<0.05), ** (p<0.01) and *** (p<0.001).
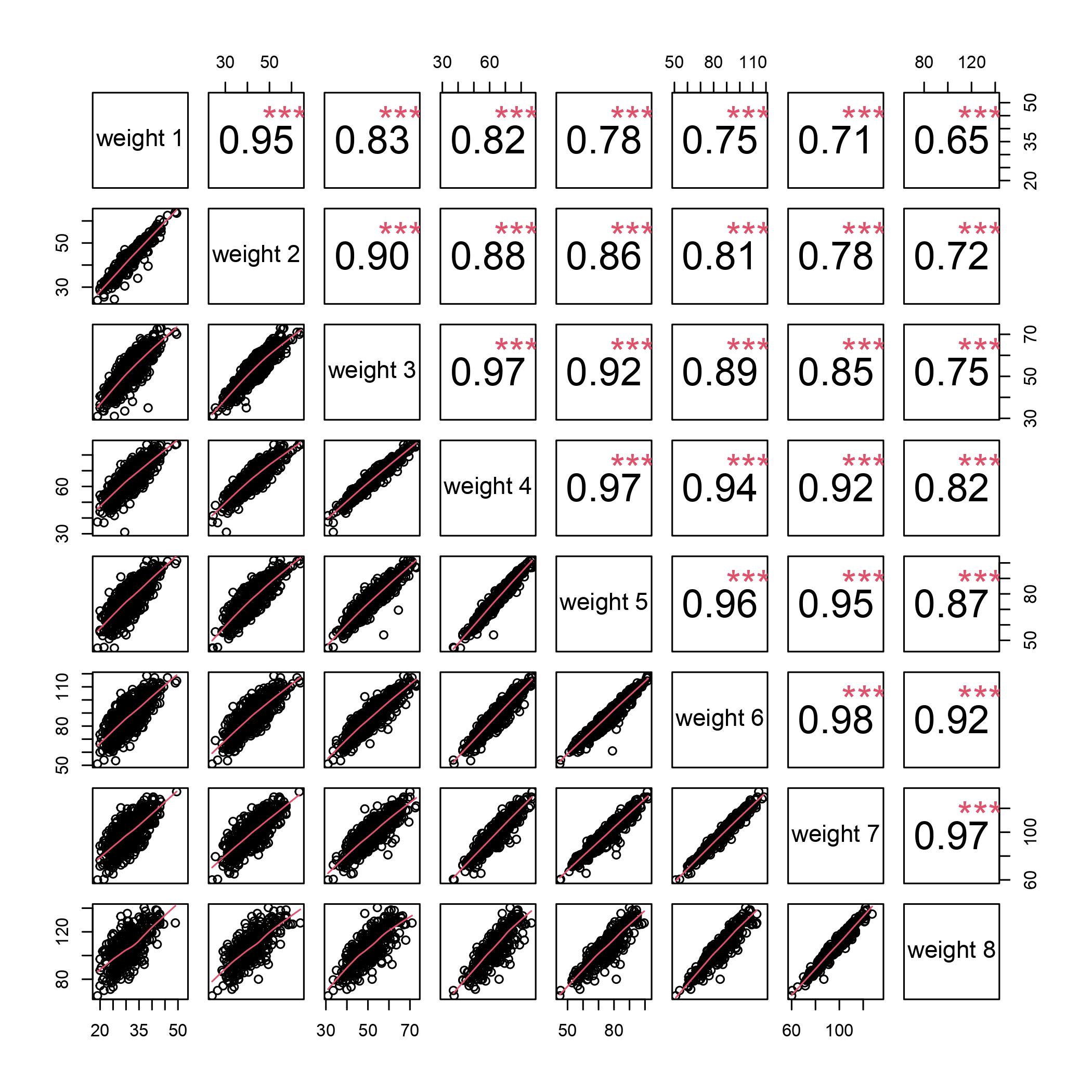

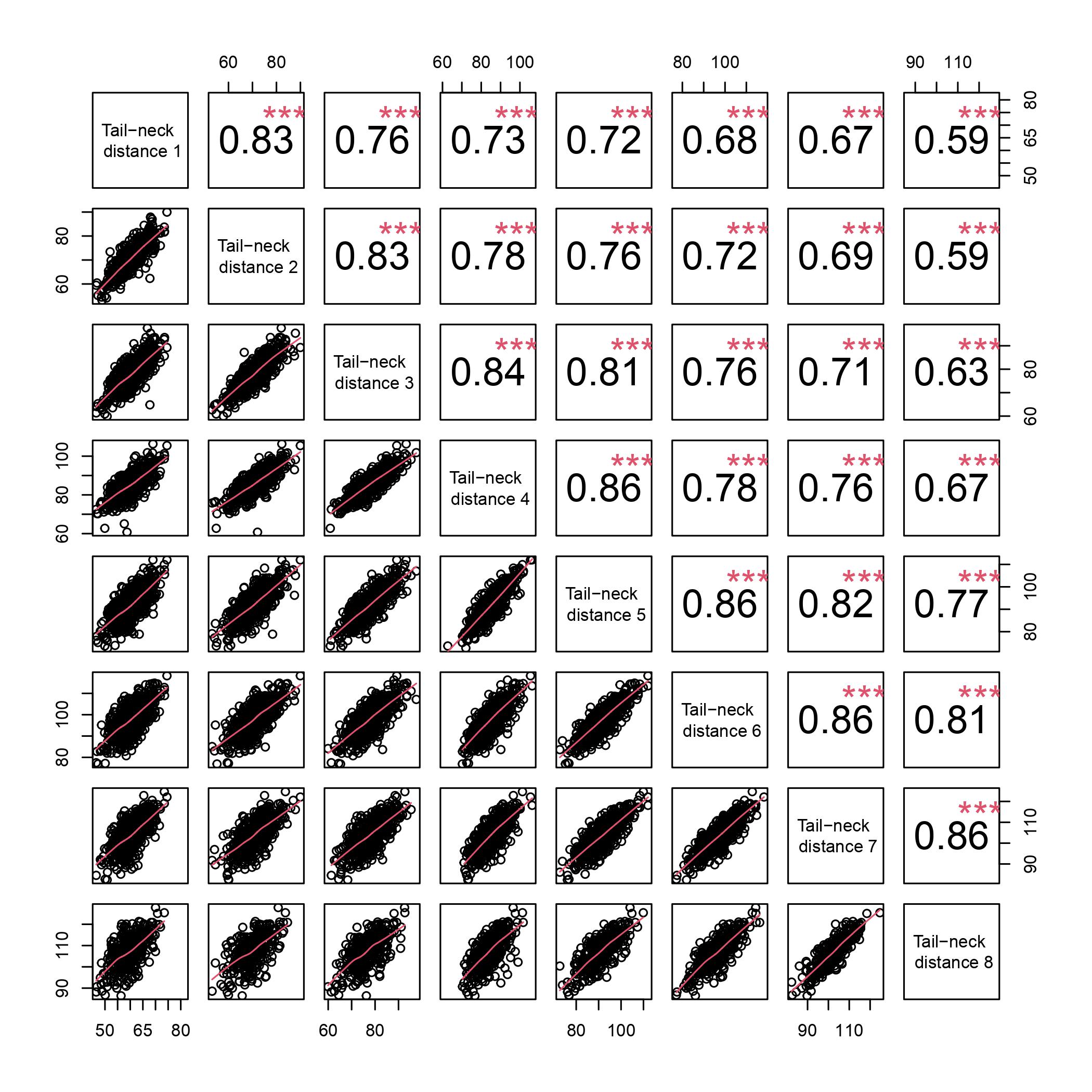

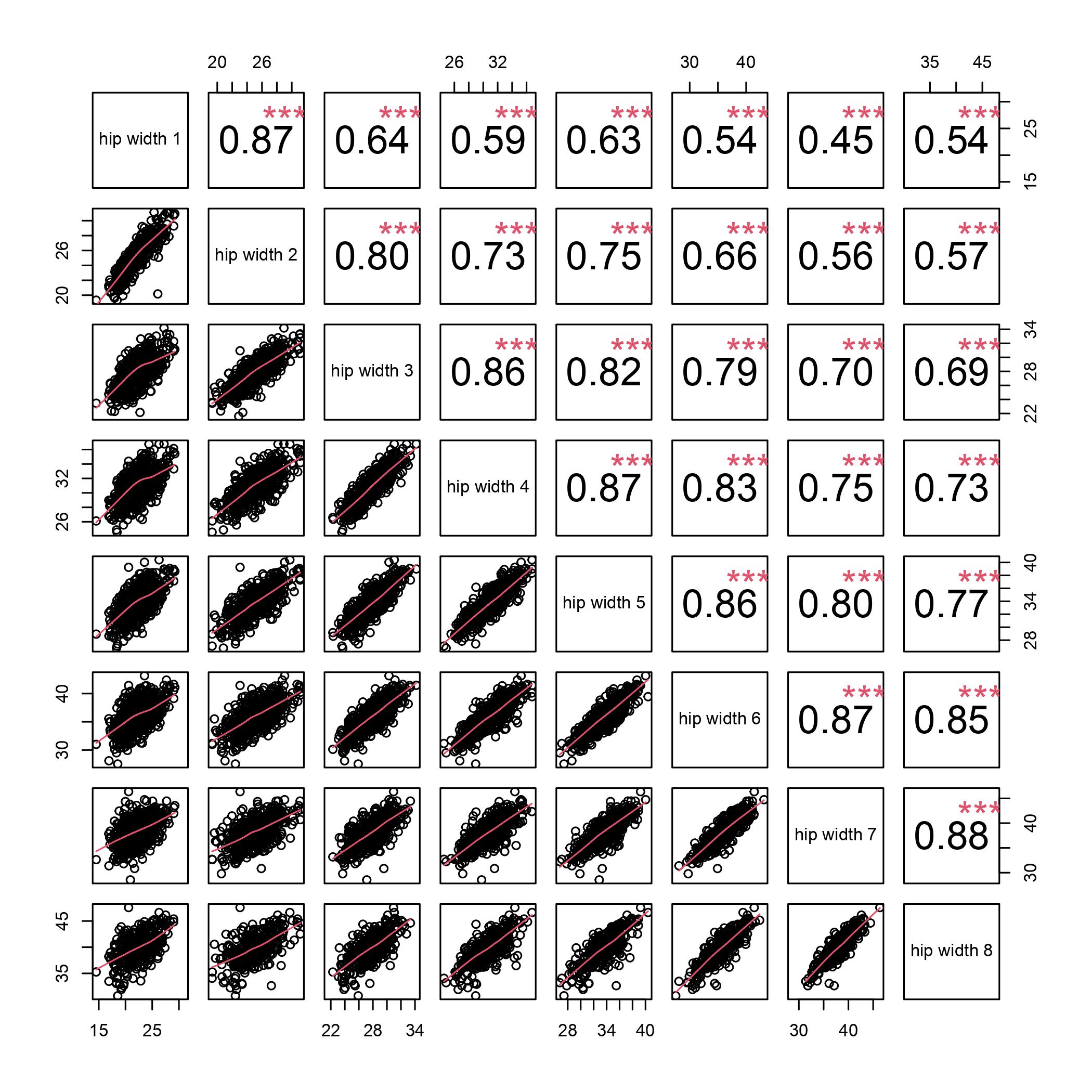

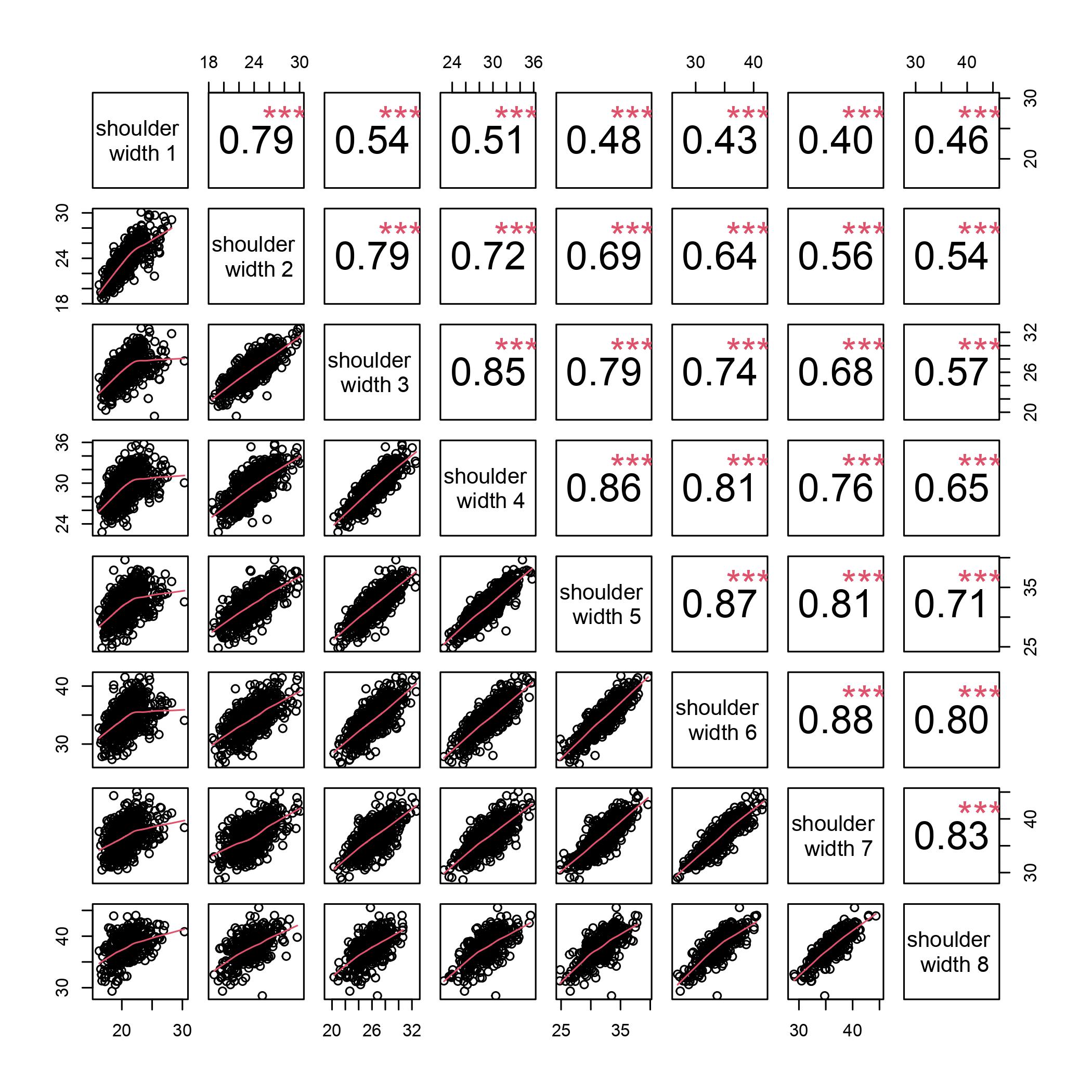

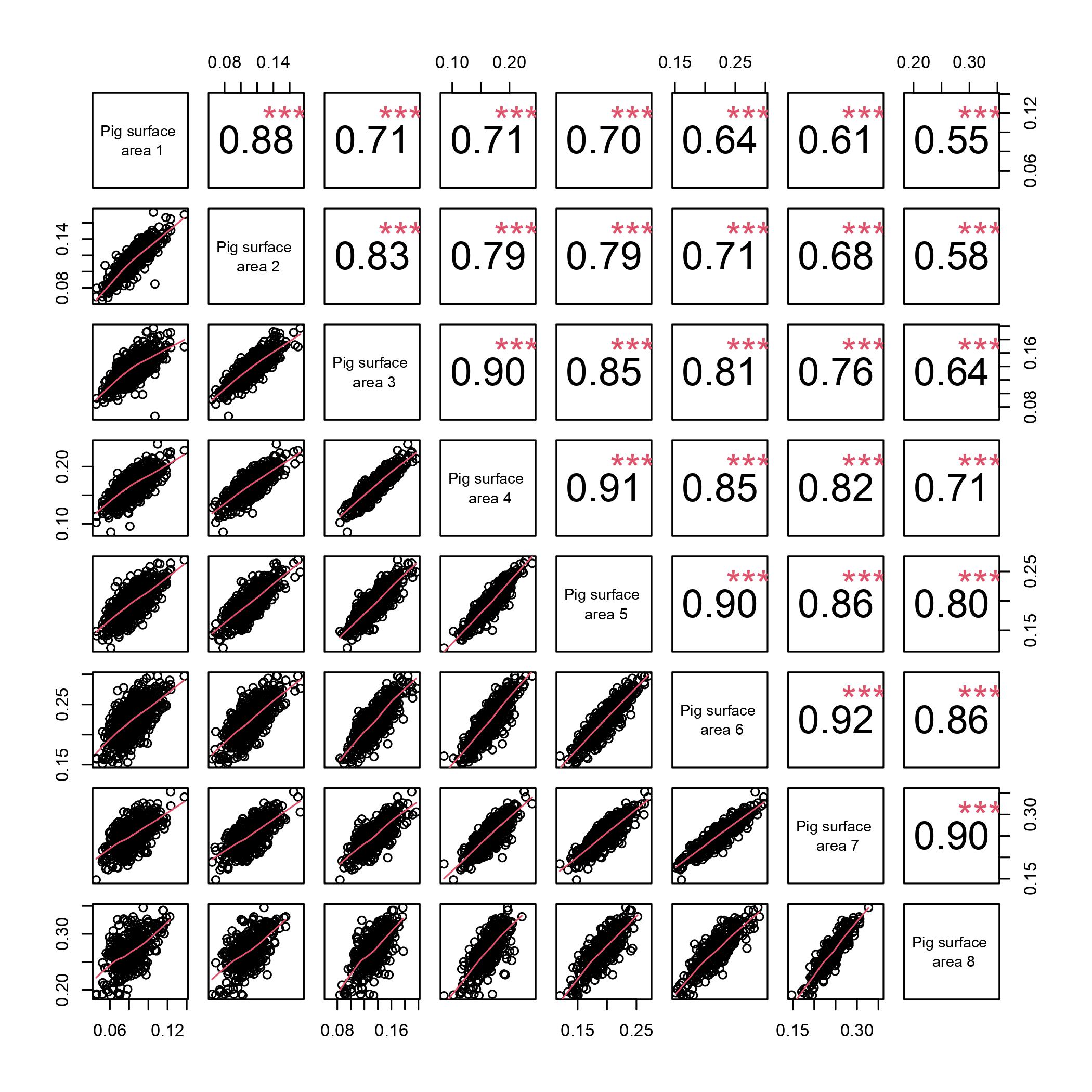

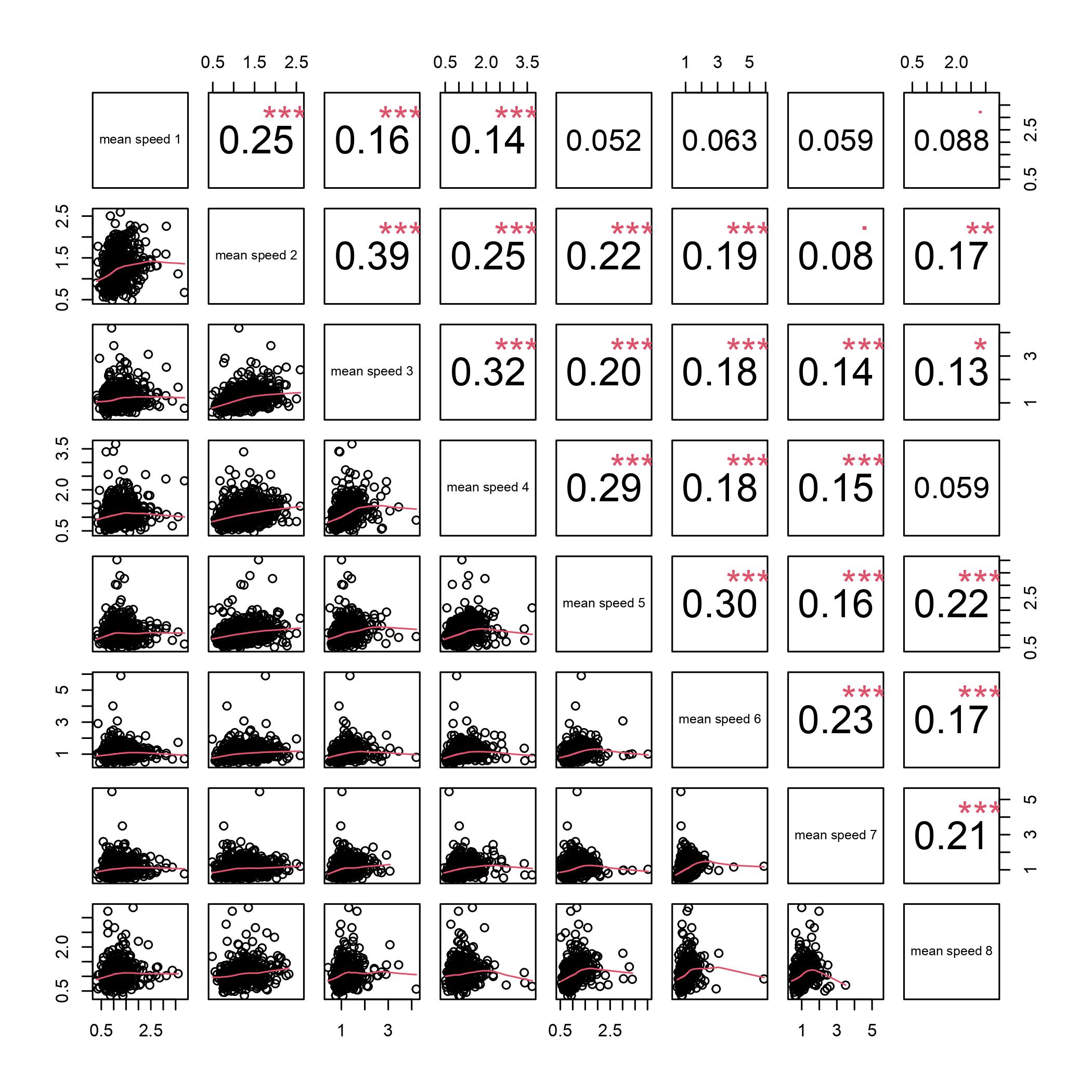

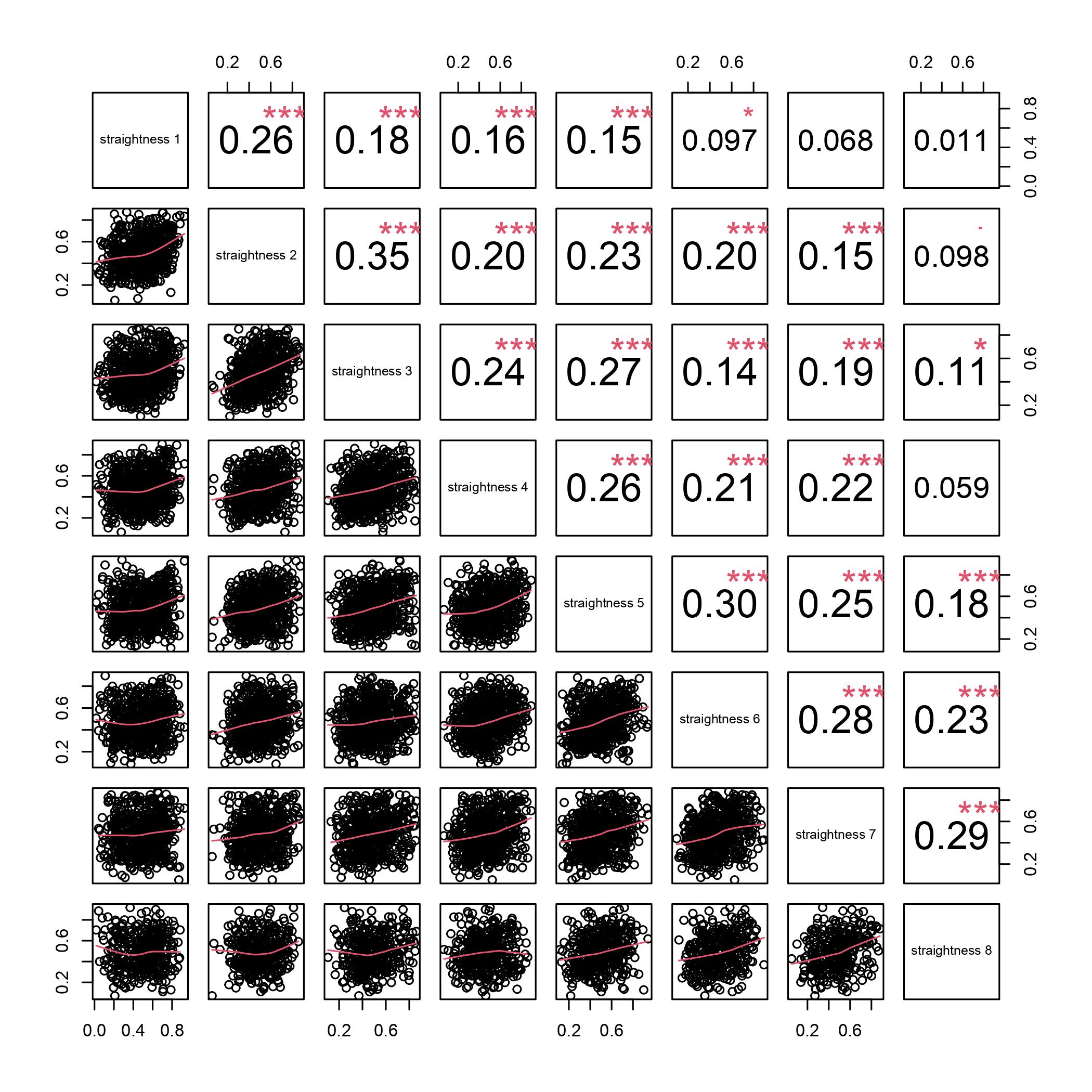

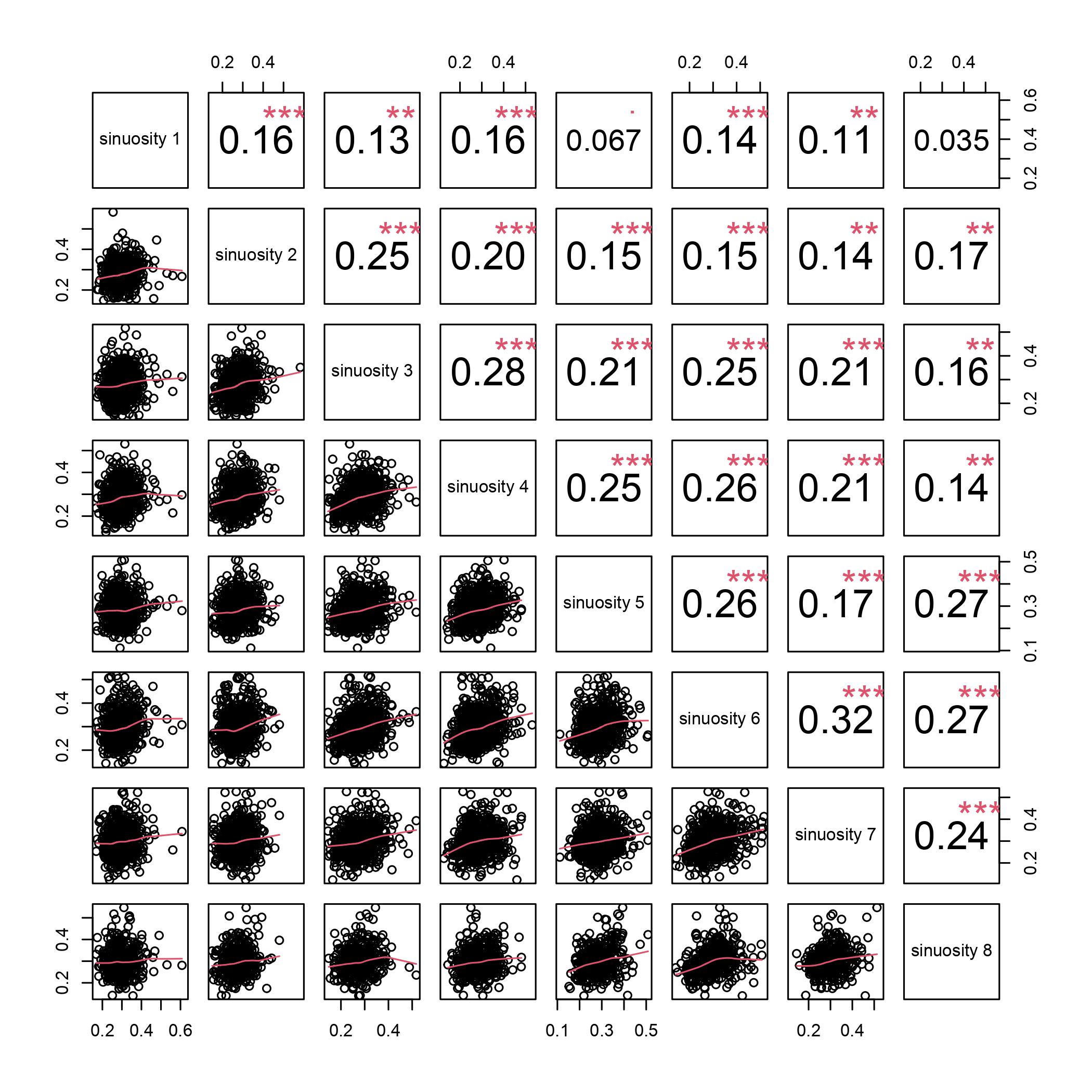

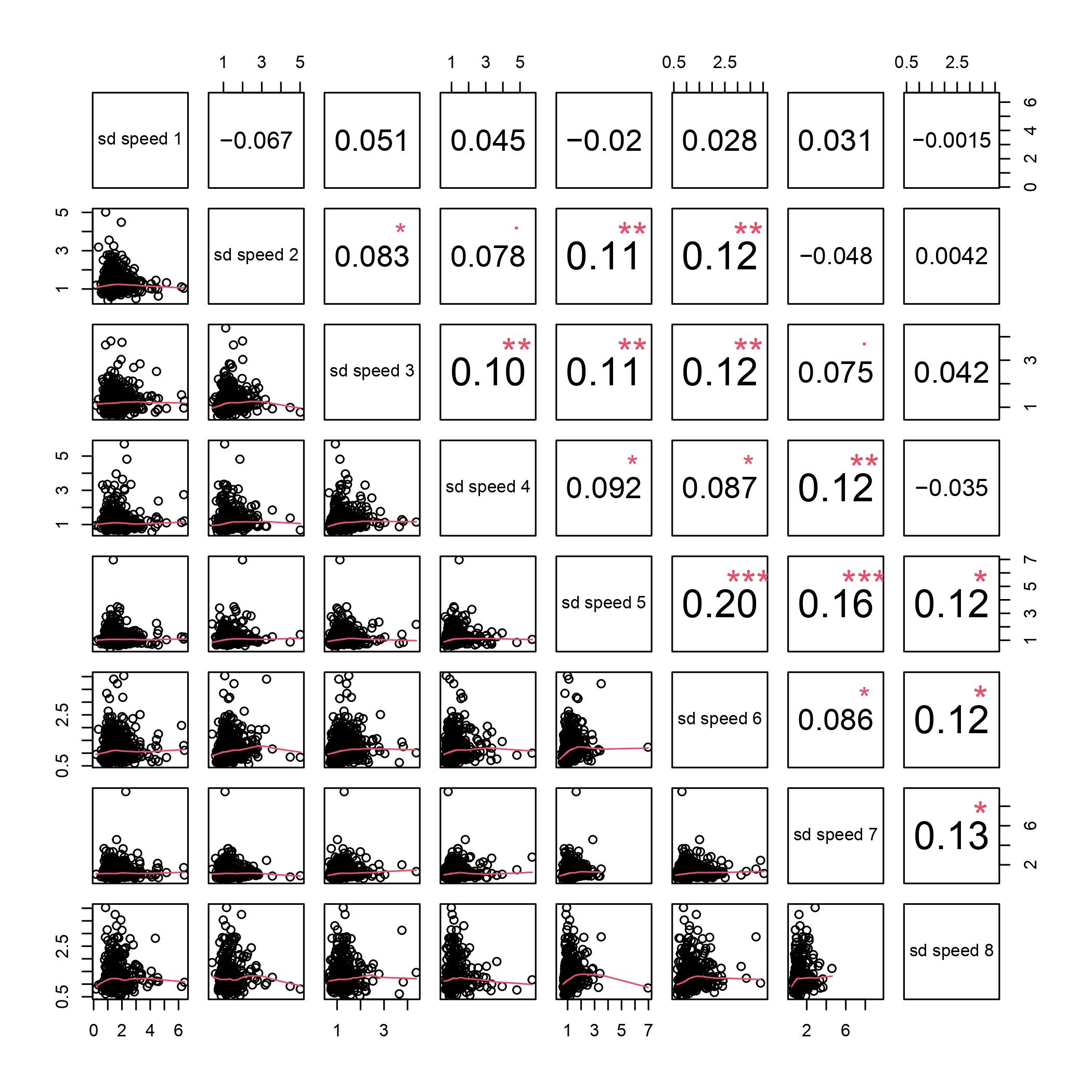

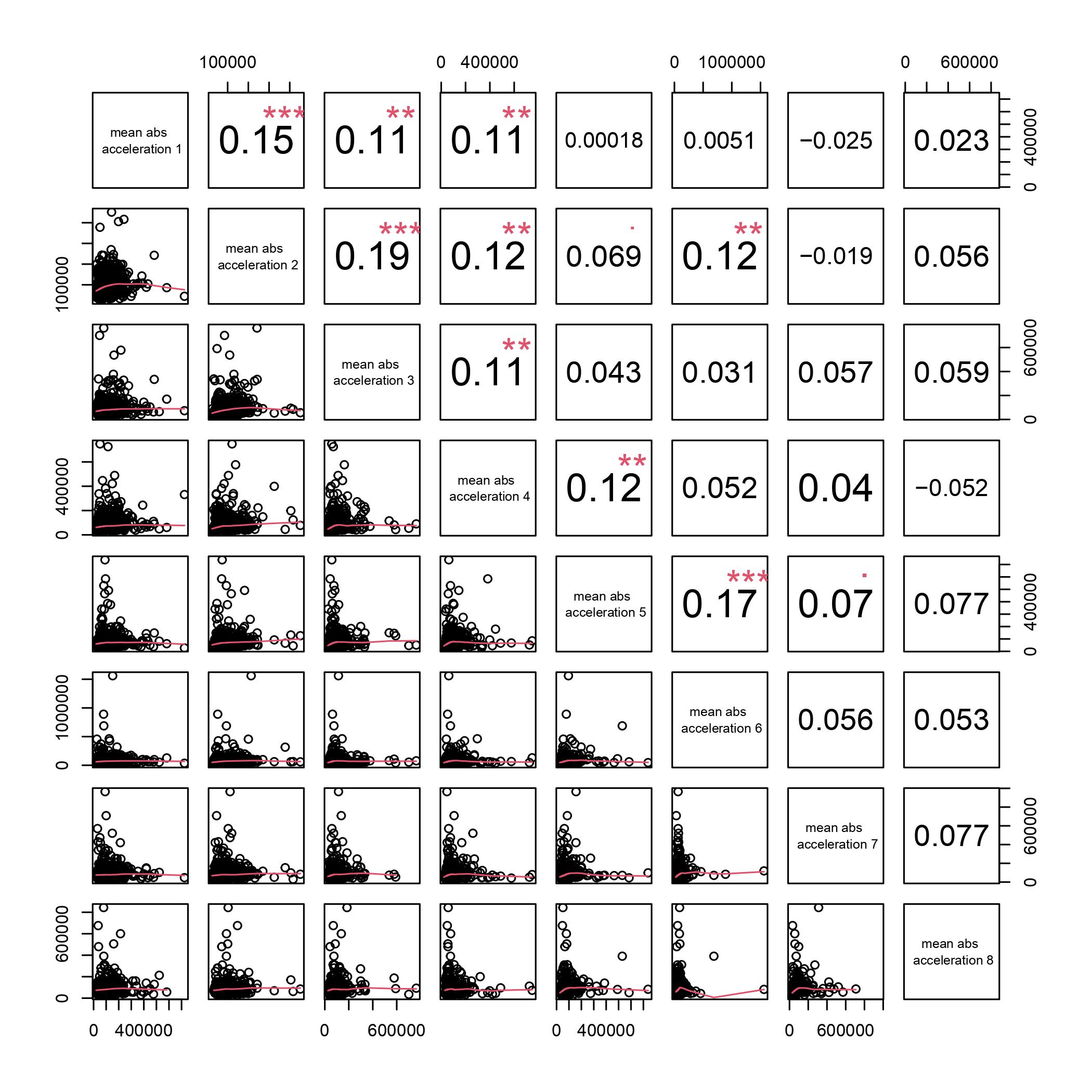

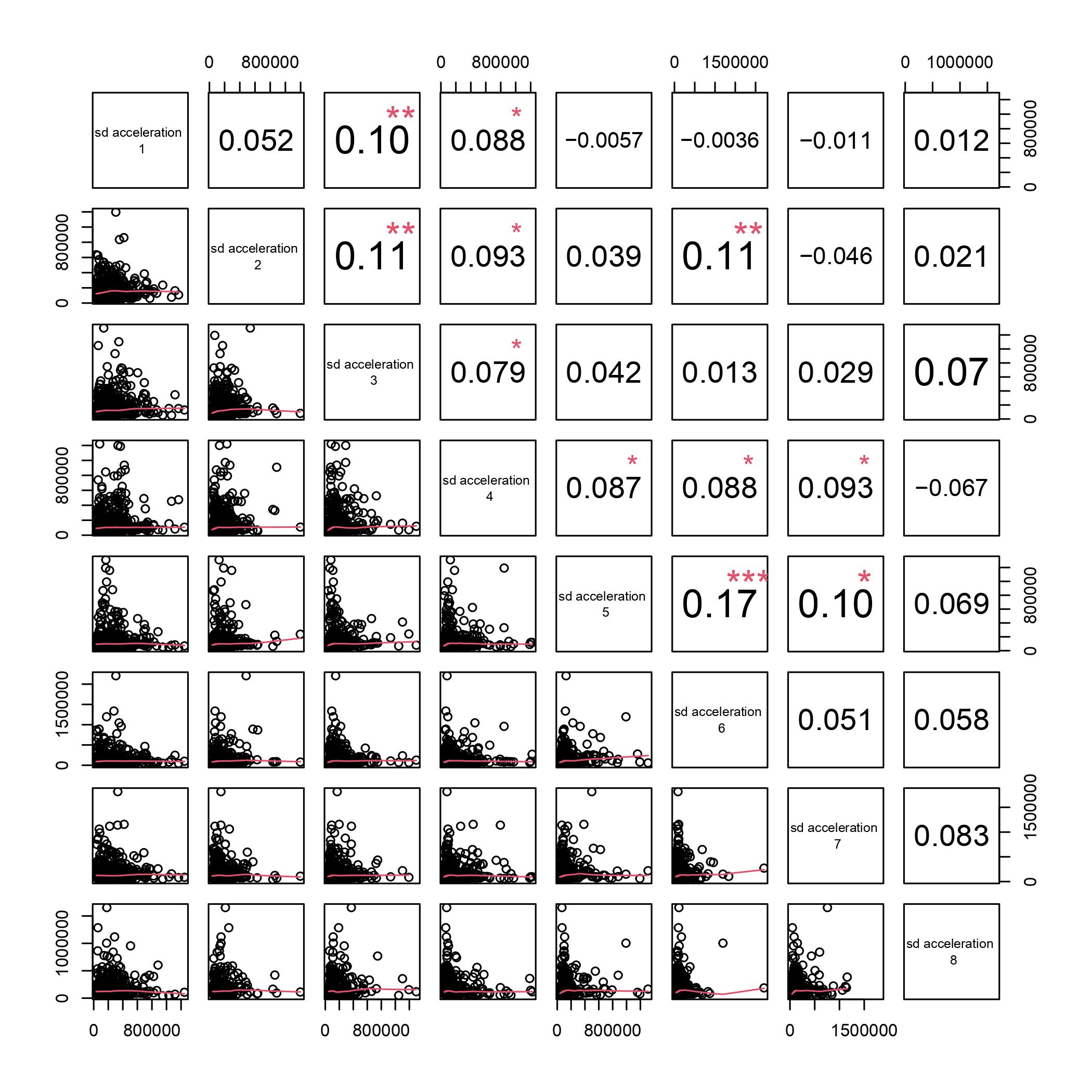
**

# **Additional File 6 Figure S3. Boxplot of heritability estimates for pigs’ body dimension and activity traits**

**Boxplots of heritability estimates (in %) are provided for a range of traits. Boxplots contain heritability estimates per weighing event (“1” to “8”) and for all data (“all”). (a) Boxplot of heritability estimates for average daily gain (ADG) and body dimension traits. (b) Boxplot of heritability estimates for automated activity traits.**

**
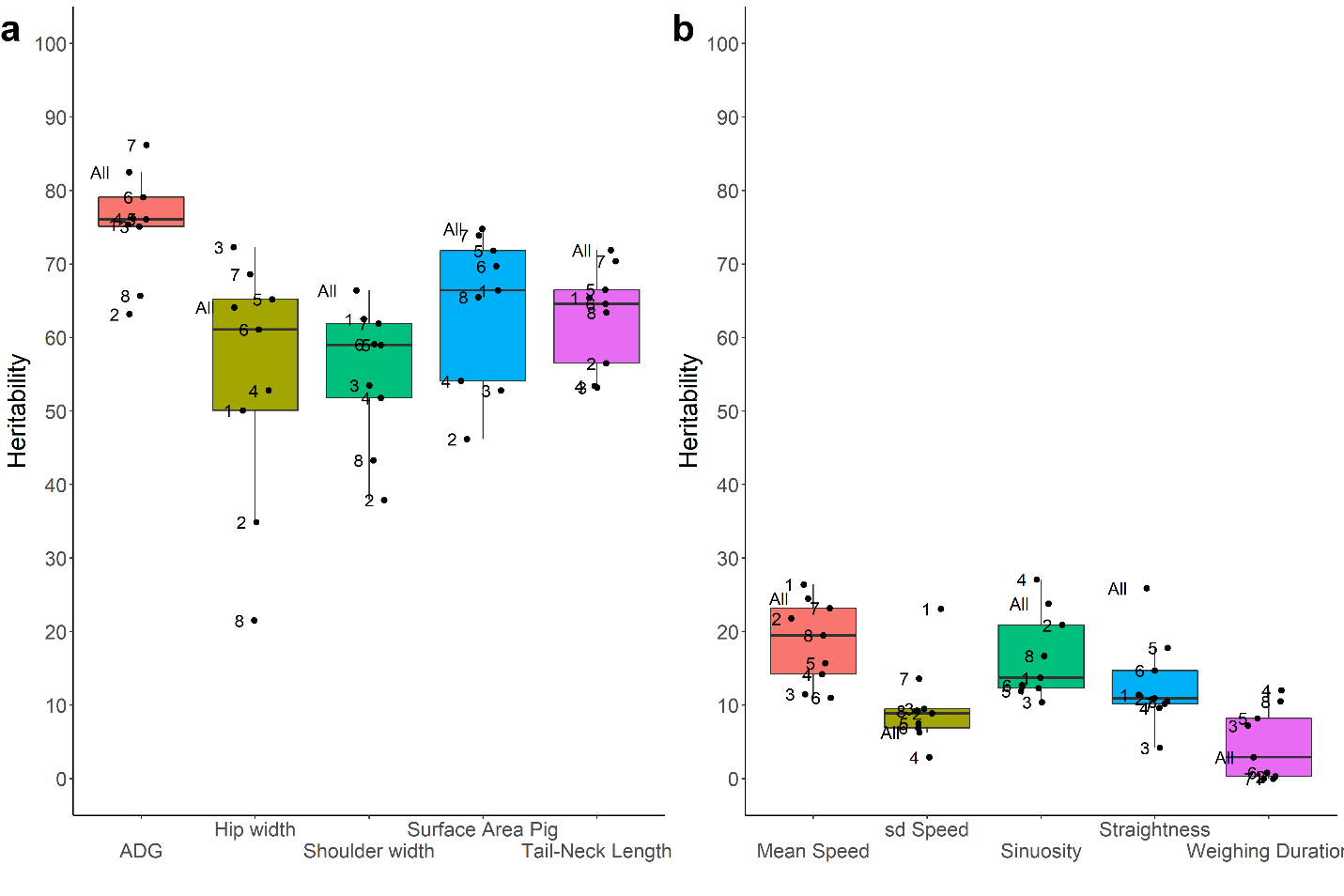
**

# **Additional File 7 Table S1. Genetic parameter estimates from univariate genetic analyses**

**Estimates of heritability (h^2^) and common environmental effects (c^2^), as well as their standard errors (SE_h^2^ and SE_c^2^) are expressed as percentage. Additive genetic standard deviation (sd_a), common environmental standard deviation (sd_c) and residual standard deviation (sd_e) are provided as well.**

| Trait | h^2^ | SE_h^2^ | c^2^ | SE_c^2^ | sd_a | sd_c | sd_e |
| --- | --- | --- | --- | --- | --- | --- | --- |
| duration_weighing (s) | 2,9 | 0,8 | 2,5 | 0,6 | 1,384 | 1,290 | 7,933 |
| sd_acceleration_Tail_base (m/s2) | 5,5 | 1 | 5,1 | 1,1 | 3,581 | 3,474 | 14,486 |
| sd_speed_Tail_base (m/s) | 6,3 | 1 | 6,6 | 1,3 | 0,133 | 0,136 | 0,494 |
| mean_abs_acceleration_Tail_base (m/s2) | 8,7 | 1,2 | 3,8 | 0,9 | 2,084 | 1,380 | 6,613 |
| straightness_index_Shoulder_right | 21,9 | 1,6 | 0,9 | 0,3 | 0,075 | 0,016 | 0,141 |
| straightness_index_Shoulder_left | 22,7 | 1,6 | 1,9 | 0,5 | 0,077 | 0,022 | 0,140 |
| sinuosity_Shoulder_right | 23,2 | 1,5 | 1,4 | 0,4 | 0,034 | 0,008 | 0,061 |
| sinuosity_Tail_base | 23,8 | 1,6 | 1,7 | 0,5 | 0,030 | 0,008 | 0,053 |
| mean_speed_Tail_base (m/s) | 24,5 | 1,6 | 2,2 | 0,6 | 0,201 | 0,061 | 0,347 |
| sinuosity_Hip_left | 25,5 | 1,6 | 2,7 | 0,6 | 0,037 | 0,012 | 0,062 |
| sinuosity_Hip_right | 25,5 | 1,6 | 2,4 | 0,6 | 0,035 | 0,011 | 0,059 |
| straightness_index_Tail_base | 25,9 | 1,6 | 0,8 | 0,3 | 0,089 | 0,015 | 0,149 |
| sinuosity_Shoulder_left | 26,6 | 1,6 | 1,8 | 0,5 | 0,039 | 0,010 | 0,063 |
| straightness_index_Hip_right | 26,6 | 1,6 | 0,9 | 0,3 | 0,090 | 0,017 | 0,149 |
| straightness_index_Hip_left | 27,9 | 1,6 | 0,9 | 0,3 | 0,093 | 0,016 | 0,149 |
| Feed intake (kg) | 32,3 | 7,8 | 67,6 | 7,8 | 0,073 | 0,106 | 0,004 |
| mean_speed_Shoulder_right | 33,8 | 1,6 | 2 | 0,5 | 20,205 | 4,879 | 27,829 |
| mean_speed_Shoulder_left | 34,6 | 1,6 | 3,8 | 0,8 | 20,910 | 6,933 | 27,920 |
| mean_speed_Hip_right | 35,4 | 1,6 | 2,5 | 0,6 | 21,120 | 5,621 | 27,989 |
| mean_speed_Hip_left | 35,6 | 1,6 | 2,5 | 0,6 | 22,031 | 5,829 | 29,021 |
| Feed Conversion_Ratio (kg/kg) | 53,4 | 8 | 46,5 | 8 | 0,060 | 0,056 | 0,003 |
| hip_width (cm) | 64,1 | 2,6 | 17,6 | 3,1 | 1,982 | 1,040 | 1,058 |
| shoulder_width (cm) | 66,4 | 1,8 | 10,8 | 1,9 | 1,967 | 0,793 | 1,154 |
| Lean Meat Percentage (%) | 69,8 | 8,2 | 4,3 | 2,8 | 2,185 | 0,542 | 1,332 |
| distance_tail_neck (cm) | 71,9 | 1,5 | 7,7 | 1,4 | 5,604 | 1,840 | 2,985 |
| Pig surface area (m2) | 74,8 | 0,8 | 2,9 | 0,1 | 0,020 | 0,004 | 0,011 |
| weight (kg) | 79,4 | 0,8 | 2,1 | 0,5 | 8,134 | 1,337 | 3,925 |
| Average Daily Gain (kg/day) | 82,5 | 1,4 | 7,7 | 1,4 | 0,065 | 0,020 | 0,022 |

# **Additional File 8 Table S2. Genetic correlation estimates from bivariate genetic analyses**

**Estimates of genetic correlations as well as standard errors using bivariate genetic animal models are given in the table below.**

| Trait1 | Trait2 | genetic_correlation | SE_genetic_correlation |
| --- | --- | --- | --- |
| duration_weighing | Pig_surface_area | 0,29 | 0,1 |
| duration_weighing | mean_speed | 0,27 | 0,12 |
| duration_weighing | sd_speed | -0,07 | 0,16 |
| duration_weighing | mean_abs_acceleration | 0,51 | 0,12 |
| duration_weighing | sd_acceleration | 0,57 | 0,13 |
| duration_weighing | Straigthness_index | -0,4 | 0,1 |
| duration_weighing | sinuosity | 0 | 0,12 |
| duration_weighing | Percentage_Lean_Meat | 0,18 | 0,41 |
| duration_weighing | Feed Conversion Ratio | 0,01 | 0,02 |
| duration_weighing | Feed Intake | 0,02 | 0,02 |
| duration_weighing | hip_width | 0,17 | 0,1 |
| duration_weighing | shoulder_width | 0,3 | 0,09 |
| duration_weighing | Tail-neck_length | 0,31 | 0,11 |
| duration_weighing | Average_Daily_Gain | 0,2 | 0,1 |
| duration_weighing | Tailbiting | 0,17 | 0,12 |
| duration_weighing | Earbiting | 0,29 | 0,13 |
| duration_weighing | EarInfection | 0,16 | 0,1 |
| Pig_surface_area | mean_speed | 0,07 | 0,04 |
| Pig_surface_area | sd_speed | -0,1 | 0,06 |
| Pig_surface_area | mean_abs_acceleration | 0,06 | 0,06 |
| Pig_surface_area | sd_acceleration | 0,03 | 0,07 |
| Pig_surface_area | Straigthness_index | -0,24 | 0,04 |
| Pig_surface_area | sinuosity | -0,09 | 0,04 |
| Pig_surface_area | Percentage_Lean_Meat | -0,44 | 0,13 |
| Pig_surface_area | Feed Conversion Ratio | -0,06 | 0,04 |
| Pig_surface_area | Feed Intake | 0,25 | 0,04 |
| Pig_surface_area | hip_width | 0,86 | 0,01 |
| Pig_surface_area | shoulder_width | 0,88 | 0,01 |
| Pig_surface_area | Tail-neck_length | 0,93 | 0 |
| Pig_surface_area | Average_Daily_Gain | 0,92 | 0,01 |
| Pig_surface_area | Tailbiting | 0,01 | 0,04 |
| Pig_surface_area | Earbiting | -0,06 | 0,04 |
| Pig_surface_area | EarInfection | -0,09 | 0,03 |
| mean_speed | sd_speed | 0,58 | 0,06 |
| mean_speed | mean_abs_acceleration | 0,92 | 0,02 |
| mean_speed | sd_acceleration | 0,77 | 0,04 |
| mean_speed | Straigthness_index | -0,93 | 0,02 |
| mean_speed | sinuosity | -0,84 | 0,02 |
| mean_speed | Percentage_Lean_Meat | -0,03 | 1,45 |
| mean_speed | Feed Conversion Ratio | -0,02 | 0,02 |
| mean_speed | Feed Intake | 0 | 0,02 |
| mean_speed | hip_width | -0,12 | 0,04 |
| mean_speed | shoulder_width | 0,04 | 0,04 |
| mean_speed | Tail-neck_length | 0,19 | 0,04 |
| mean_speed | Average_Daily_Gain | -0,09 | 0,04 |
| mean_speed | Tailbiting | -0,04 | 0,05 |
| mean_speed | Earbiting | 0,03 | 0,06 |
| mean_speed | EarInfection | -0,03 | 0,04 |
| sd_speed | mean_abs_acceleration | 0,64 | 0,06 |
| sd_speed | sd_acceleration | 0,65 | 0,07 |
| sd_speed | Straigthness_index | -0,46 | 0,08 |
| sd_speed | sinuosity | -0,38 | 0,08 |
| sd_speed | Percentage_Lean_Meat | -0,07 | 0,4 |
| sd_speed | Feed Conversion Ratio | -0,02 | 0,02 |
| sd_speed | Feed Intake | 0,12 | 0,02 |
| sd_speed | hip_width | -0,09 | 0,07 |
| sd_speed | shoulder_width | -0,14 | 0,07 |
| sd_speed | Tail-neck_length | -0,05 | 0,07 |
| sd_speed | Average_Daily_Gain | -0,16 | 0,06 |
| sd_speed | Tailbiting | -0,09 | 0,08 |
| sd_speed | Earbiting | -0,02 | 0,09 |
| sd_speed | EarInfection | -0,06 | 0,07 |
| mean_abs_acceleration | sd_acceleration | 0,95 | 0,01 |
| mean_abs_acceleration | Straigthness_index | -0,86 | 0,04 |
| mean_abs_acceleration | sinuosity | -0,61 | 0,06 |
| mean_abs_acceleration | Percentage_Lean_Meat | 0,02 | 0,37 |
| mean_abs_acceleration | Feed Conversion Ratio | 0,64 | 0,01 |
| mean_abs_acceleration | Feed Intake | 0,03 | 0,02 |
| mean_abs_acceleration | hip_width | -0,11 | 0,06 |
| mean_abs_acceleration | shoulder_width | 0,06 | 0,06 |
| mean_abs_acceleration | Tail-neck_length | 0,18 | 0,06 |
| mean_abs_acceleration | Average_Daily_Gain | -0,1 | 0,06 |
| mean_abs_acceleration | Tailbiting | -0,02 | 0,07 |
| mean_abs_acceleration | Earbiting | 0,06 | 0,08 |
| mean_abs_acceleration | EarInfection | -0,03 | 0,06 |
| sd_acceleration | Straigthness_index | -0,73 | 0,05 |
| sd_acceleration | sinuosity | -0,44 | 0,08 |
| sd_acceleration | Percentage_Lean_Meat | 0,11 | 0,38 |
| sd_acceleration | Feed Conversion Ratio | 0 | 0,02 |
| sd_acceleration | Feed Intake | 0,03 | 0,02 |
| sd_acceleration | hip_width | -0,08 | 0,07 |
| sd_acceleration | shoulder_width | 0,07 | 0,07 |
| sd_acceleration | Tail-neck_length | 0,14 | 0,07 |
| sd_acceleration | Average_Daily_Gain | -0,09 | 0,07 |
| sd_acceleration | Tailbiting | 0,01 | 0,08 |
| sd_acceleration | Earbiting | 0,12 | 0,09 |
| sd_acceleration | EarInfection | -0,02 | 0,07 |
| Straigthness_index | sinuosity | 0,87 | 0,03 |
| Straigthness_index | Percentage_Lean_Meat | -0,13 | 0,4 |
| Straigthness_index | Feed Conversion Ratio | 0,06 | 0,02 |
| Straigthness_index | Feed Intake | -0,02 | 0,02 |
| Straigthness_index | hip_width | -0,02 | 0,04 |
| Straigthness_index | shoulder_width | -0,17 | 0,04 |
| Straigthness_index | Tail-neck_length | -0,34 | 0,04 |
| Straigthness_index | Average_Daily_Gain | -0,05 | 0,04 |
| Straigthness_index | Tailbiting | -0,02 | 0,05 |
| Straigthness_index | Earbiting | -0,08 | 0,06 |
| Straigthness_index | EarInfection | -0,07 | 0,04 |
| sinuosity | Percentage_Lean_Meat | 0,21 | 0,26 |
| sinuosity | Feed Conversion Ratio | 0,03 | 0,02 |
| sinuosity | Feed Intake | 0,03 | 0,02 |
| sinuosity | hip_width | 0,07 | 0,04 |
| sinuosity | shoulder_width | -0,02 | 0,04 |
| sinuosity | Tail-neck_length | -0,18 | 0,04 |
| sinuosity | Average_Daily_Gain | 0,07 | 0,04 |
| sinuosity | Tailbiting | 0,04 | 0,05 |
| sinuosity | Earbiting | 0,02 | 0,06 |
| sinuosity | EarInfection | 0,02 | 0,04 |
| Percentage_Lean_Meat | Feed Conversion Ratio | 0 | 0,02 |
| Percentage_Lean_Meat | Feed Intake | -0,14 | 0,02 |
| Percentage_Lean_Meat | hip_width | -0,46 | 0,14 |
| Percentage_Lean_Meat | shoulder_width | -0,34 | 0,15 |
| Percentage_Lean_Meat | Tail-neck_length | -0,35 | 0,14 |
| Percentage_Lean_Meat | Average_Daily_Gain | -0,49 | 0,11 |
| Percentage_Lean_Meat | Tailbiting | -0,03 | 0,15 |
| Percentage_Lean_Meat | EarInfection | 0,38 | 0,52 |
| Feed Conversion Ratio | Feed Intake | 0,08 | 0,02 |
| Feed Conversion Ratio | hip_width | -0,05 | 0,02 |
| Feed Conversion Ratio | shoulder_width | -0,06 | 0,02 |
| Feed Conversion Ratio | Tail-neck_length | -0,07 | 0,02 |
| Feed Conversion Ratio | Average_Daily_Gain | -0,05 | 0,02 |
| Feed Conversion Ratio | Tailbiting | 0,03 | 0,02 |
| Feed Conversion Ratio | Earbiting | 0,01 | 0,02 |
| Feed Conversion Ratio | EarInfection | 0,04 | 0,02 |
| Feed Intake | hip_width | 0,22 | 0,01 |
| Feed Intake | shoulder_width | 0,22 | 0,01 |
| Feed Intake | Tail-neck_length | 0,21 | 0,02 |
| Feed Intake | Average_Daily_Gain | 0,31 | 0,01 |
| Feed Intake | Tailbiting | -0,12 | 0,02 |
| Feed Intake | Earbiting | 0,03 | 0,02 |
| Feed Intake | EarInfection | -0,02 | 0,02 |
| hip_width | shoulder_width | 0,83 | 0,01 |
| hip_width | Tail-neck_length | 0,67 | 0,02 |
| hip_width | Average_Daily_Gain | 0,9 | 0,01 |
| hip_width | Tailbiting | -0,06 | 0,04 |
| hip_width | Earbiting | -0,03 | 0,04 |
| hip_width | EarInfection | -0,07 | 0,03 |
| shoulder_width | Tail-neck_length | 0,7 | 0,02 |
| shoulder_width | Average_Daily_Gain | 0,87 | 0,01 |
| shoulder_width | Tailbiting | -0,01 | 0,04 |
| shoulder_width | Earbiting | -0,03 | 0,04 |
| shoulder_width | EarInfection | -0,08 | 0,03 |
| Tail-neck_length | Average_Daily_Gain | 0,81 | 0,01 |
| Tail-neck_length | Tailbiting | 0,03 | 0,04 |
| Tail-neck_length | Earbiting | -0,06 | 0,04 |
| Tail-neck_length | EarInfection | -0,09 | 0,03 |
| Average_Daily_Gain | Tailbiting | -0,02 | 0,04 |
| Average_Daily_Gain | Earbiting | -0,05 | 0,04 |
| Average_Daily_Gain | EarInfection | -0,1 | 0,03 |
| Tailbiting | Earbiting | 0,35 | 0,06 |
| Tailbiting | EarInfection | 0 | 0,04 |
| Earbiting | EarInfection | -0,05 | 0,04 |
